# *FCclasses3*: Vibrationally‐resolved spectra simulated at the edge of the harmonic approximation

**DOI:** 10.1002/jcc.27027

**Published:** 2022-11-15

**Authors:** Javier Cerezo, Fabrizio Santoro

**Affiliations:** ^1^ Departamento de Química and Institute for Advanced Research in Chemical Sciences (IAdChem) Universidad Autónoma de Madrid Madrid Spain; ^2^ Consiglio Nazionale delle Ricerche Istituto di Chimica dei Composti Organo Metallici (ICCOM‐CNR) Pisa Italy

**Keywords:** circularly polarized luminescence, curvilinear internal coordinates, electronic circular dichroism, linear and nonlinear, magnetic circular dichroism, nonradiative rates, one‐photon absorption, time‐independent and time‐dependent, vibronic spectroscopy

## Abstract

We introduce *FCclasses3*, a code to carry out vibronic simulations of electronic spectra and nonradiative rates, based on the harmonic approximation. Key new features are: implementation of the full family of vertical and adiabatic harmonic models, vibrational analysis in curvilinear coordinates, extension to several electronic spectroscopies and implementation of time‐dependent approaches. The use of curvilinear valence internal coordinates allows the adoption of quadratic model potential energy surfaces (PES) of the initial and final states expanded at arbitrary configurations. Moreover, the implementation of suitable projectors provides a robust framework for defining reduced‐dimensionality models by sorting flexible coordinates out of the harmonic subset, so that they can then be treated at anharmonic level, or with mixed quantum classical approaches. A set of tools to facilitate input preparation and output analysis is also provided. We show the program at work in the simulation of different spectra (one and two‐photon absorption, emission and resonance Raman) and internal conversion rate of a typical rigid molecule, anthracene. Then, we focus on absorption and emission spectra of a series of flexible polyphenyl molecules, highlighting the relevance of some of the newly implemented features. The code is freely available at http://www.iccom.cnr.it/en/fcclasses/.

## Graphical abstract

*FCclasses3* is a code to simulate vibronic spectra and nonradiative rates based on the harmonic approximation, supporting a wide range of spectroscopies. It makes freely available to the community the developments carried out in our group in the last 10 years, including time‐dependent formulations in internal coordinates, which brings us to the limits of the harmonic approximation. It allows users to easily compute different properties and gives access to advanced features through a simple and flexible input.

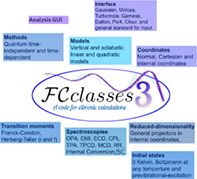

## 1 INTRODUCTION

Electronic spectroscopy has ubiquitous applications in current research ranging from chemistry to materials science and biology. Different electronic spectroscopies investigate different properties of the system and in all cases the shape and position of the spectra arise from the interplay of intra‐molecular and/or inter‐molecular factors whose individual role cannot be easily extracted just from the inspection of the experimental trace. Thanks to recent progresses, computational spectroscopy stands nowadays as a mature and robust approach to complement the experimental measurements and help retrieving all possible information entailed in the spectroscopic signals.^1^ It is well assessed that in order to describe the shape of the electronic spectra it is necessary to adopt theoretical models which explicitly address the coupling of the electronic transition with the nuclear motion (vibronic transitions).^2^ The same is true for the simulation of the rates on nonradiative processes. The degree of complexity of the just formulated problem can be formidable, making it impossible finding a general solution.

Here we focus on the class of relatively large systems (dozens or hundreds of vibrational coordinates) which are rigid or with few flexible degrees of freedom (DoFs), so that harmonic approximation represents a useful framework to describe their PES, and whose electronic states involved in the radiative transition exhibit negligible or weak inter‐state couplings. The field of the simulation of the vibronic spectroscopy of these systems has made remarkable progress in the last two decades thanks to the contributions of several groups, which is impossible to review here (see for instance the review papers in Refs. 2, 3, 4). Our group has been active in this context and in this contribution, we present *FCclasses3*, the new and largely renovated and extended release of our code *FCclasses* distributed in 2009, which makes freely available all the computational methods we have developed in the last decade in the field of vibronic spectroscopy.

As we mentioned above our computational approaches are based on the hypothesis that initial and final electronic states are not significantly coupled to any other state. Otherwise, the solution can be obtained through either time‐independent approaches, like the Lanczos method^5^, ^6^, ^7^ or numerical quantum dynamics propagation of the wavepacket over the coupled states.^5^ Moreover, all PES are described within harmonic approximation, which is generally valid for stiff modes but inadequate to account for flexible and/or very anharmonic regions of the PES. The coupling triggering the transition between the initial and final states is generally considered small enough so that a Fermi Golden rule approach can be adopted. Finally, the nuclear dependence of electronic coupling terms, which can be expressed as a Taylor expansion, are usually described taking only the constant term (Franck‐Condon, FC, approximation) or up to the linear term (usually called Herzberg‐Teller, HT). The HT approximation introduces perturbatively the effect of inter‐state couplings when they are weak and the states are well separated. Whereas most of the developments reported in literature focus on linear‐spectroscopies, extension to some nonlinear spectroscopies like resonance Raman is straightforward,^8^, ^9^, ^10^ while, vibronic computations of other signals like two‐photon absorption,^11^, ^12^ circular‐dichroism^12^ or magnetic circular dichroism^13^ within this framework are still doable but they require some additional approximation on the frequency‐dependence of the transition amplitudes.

Adopting all the approximations sketched above, analytical expressions emerge for the intensities of the different spectroscopies and nonradiative processes, which can be formulated in terms of sum‐over‐states (what we will refer to as time independent [TI]) or Fourier‐transform of correlation functions (what we will refer to as time‐dependent [TD]). The basic general fundamental equations for both formulations have been known for decades.^14^, ^15^, ^16^ However, the limited availability of second derivatives of the PES, which are required for harmonic models, made scarcely useful the development of efficient computational schemes for practical computations. The derivation of analytical formulas for first and second derivatives of the excited‐state energy for different electronic structure methods, primarily the popular time‐dependent density functional theory (TD‐DFT) has completely changed the scenario in the last decades, stimulating, in the last 20 years, a fast flourishing of efficient methods for specific cases within both TI^17^, ^18^, ^19^, ^20^, ^21^ and TD^22^, ^23^, ^24^, ^25^, ^26^ formulations, and their widespread applications in literature. In the case of TI approaches, the main challenges to be overcome are related to the need to evaluate the intensity of the vibronic transitions between a vast number of possible initial and final states. This difficulty has called for the elaboration of effective filtering protocols to select only the most relevant transitions, and fast ways to compute them. Just to make an example, for a typical semi‐rigid molecule with 100 normal modes millions or even billions of FC overlaps need to be evaluated for a spectrum convergence >90% (defined as the recovered fraction of the total intensity evaluated from analytical sums). Regarding TD approaches, the major developments have gone through deriving the exact analytical expression of the correlation function for different cases (under the harmonic approximation), including one photon spectroscopies with FC and HT terms, resonance Raman,^9^, ^27^, ^28^ nonradiative process as internal conversion and intersystem crossing,^29^, ^30^ and two‐photon absorption.^31^, ^32^ It is interesting to note that TI and TD formulations are somehow complementary. TI provides full information about the actual vibronic transitions responsible for the main spectral features, but can suffer from convergence problems. TD does not provide information on the individual vibronic transitions but it delivers fully converged spectra, that is, effectively accounting for all possible transitions.

Another essential point to consider are thermal effects, which tune the distribution of initially populated states. In TI formulations, this calls for an update of the filtering method to effectively account for the additional transitions.^33^ On the contrary, in most cases TD formulations already account for temperature effects at no additional cost. It should be added that in some specific spectroscopies the electronic transition takes place from a nonthermal initial vibrational population, created by a infrared pre‐excitation, leading to the so‐called VIPER spectroscopy. This IR pre‐excitation can be accounted for in both TI and TD formulations.^34^

*FCclasses3* is a program, written in Fortran90 with legacy parts in Fortran77, to compute electronic spectra and kinetic rates of nonradiative processes, including vibrational effects at the harmonic level. The present code largely extends the capabilities of the program *FCclasses*,^35^ which was based on an efficient algorithm for the calculation of one photon spectra adopting TI formulations. Concretely, among other features, this new version implements the full family of adiabatic and vertical harmonic models and it generalizes harmonic models allowing the quadratic expansion at arbitrary geometries using either Cartesian (valid only at stationary points) or valence internal coordinates. The number of supported properties has been notably increased from the ones included in the previous release, namely one‐photon spectroscopies as one‐photon absorption (OPA) and emission (EMI) and electronic circular dichroism (ECD). *FCclasses3* can now compute additional one‐photon chiral spectroscopic techniques: Circularly polarized luminescence (CPL) and magnetic circular dichroism (MCD) as well as two‐photon spectroscopies, including absorption (TPA) and circular dichroism (TPCD), and it also supports resonance Raman (RR). Regarding nonradiative processes, it includes the calculation of internal conversion (IC) rates induced by nonadiabatic couplings and more generally of any transition between (diabatic) states driven by a constant scalar coupling (NRSC). Finally, the computation of all available properties can be performed with both TI implementations, exploiting and extending the TI algorithm present in the previous version of the code, and new TD approaches, through purposely derived analytical correlation functions.

As mentioned before, the simulation of vibronic spectra has attracted much interest in the past years. Beside the previous version of *FCclasses* (2.1), distributed since 2009, there are currently available several other codes devoted to such task such as FCBand^36^ or ezSpectrum,^37^ and it is now an add‐on included in many electronic structure programs including Gaussian16,^38^ Orca5.0,^39^ Q‐Chem,^40^ or Molpro.^41^ The important contributions in these fields by groups that implement their derivations in codes shared to different extents with the community should also be mentioned. The complete list is too vast to be listed here entirely, but, to name just a few, we can mention the contributions by Grimme,^17^ Peluso,^42^ Berger,^43^ or Nooijen.^20^ It is not the scope of the present article to make a detailed comparison of the different features of all the available codes. To the best of our knowledge, *FCclasses3* stands among the most flexible and complete packages, and it includes a number of unique features, such as the possibility to define reduced dimensionality models through the availability of projectors in curvilinear internal coordinates,^44^ the simulation of spectra starting from selected vibrationally excited states (which is the base of VIPER spectroscopy),^45^ or the availability of some more exotic properties like the two‐photon circular dichroism.^12^ Moreover, we made a particular effort to give the user precise control of the different options in the different computational protocols and feed input data in several ways to ease its use.

All methods discussed up to now are formulated adopting the harmonic approximation, limiting the applicability to relatively rigid systems. The range of validity of the harmonic approximation is however somewhat dependent on the system of coordinates adopted to describe the normal modes. Namely, the use of linear coordinates, as Cartesian, can lead to severe issues when dealing with significant displacements along curvilinear DoFs, such as torsions. In these cases, Cartesian coordinates, artificially couple low‐frequency modes (associated with such large‐amplitude displacements) with high‐frequency ones introducing large artifacts in the spectra.^46^, ^47^ When curvilinear coordinates (e.g., valence internal coordinates) are used instead, and this is possible in *FCclasses3*, such spurious couplings vanish. It has been shown that this extends the range of applicability of the harmonic approximation, preventing the occurrence of such artifacts.^48^, ^49^ It remains clear that, when significant anharmonicities of large amplitude motions come into play, they need to be dealt with specific methodologies beyond harmonic approximation.

*FCclasses3* does not compute anharmonic vibronic spectra. The literature on aharmonic vibronic computations is too broad to be reviewed here. We just mention that focusing on TI approaches, anharmonic vibrational states in very few coordinates (and the corresponding FC overlaps) can be computed with straightforward variational approaches, and many groups have developed their own house‐made codes for these tasks. When anharmonicity involves many dimensions, sophisticated approaches like the vibrational configuration interaction (VCI) or the vibrational coupled cluster (VCC),^50^, ^51^ can be applied but they require a quite different computational machinery; the interested user is referred to the implementations available in codes like Molpro^41^ or MidasCpp.^52^ Finally, also TD approaches can be profitably used for anharmonic systems, exploiting for instance the potentiality of the multiconfigurational time‐dependent Hartree method (MCTDH),^53^ and its implementation in very versatile distributed codes like Quantics.^54^

In this context, it is worthy to highlight that the use of curvilinear internal coordinates in *FCclasses3* does open the route to different approximate strategies to compute the shape of electronic spectra of flexible molecules. In fact, one side they allow the expansion of the harmonic potential also at nonstationary points of the PES and, on the other side, they provide a clean way to separate the flexible, anharmonic DoFs, thanks to the iterative projectors implemented in the code. It is therefore possible to set up mixed approaches where vibronic models in harmonic approximation are still applied to the molecular stiff modes, while flexible anharmonic ones are treated with alternative methodologies. Besides the straightforward application of variational approaches when flexibility involves very few coordinates,^55^ we here mention the Adiabatic Molecular Dynamics generalized Vertical Hessian approach (Ad‐MD|gVH) we proposed recently.^56^ In this method, which is based on *FCclasses3* and its interface with other codes, anharmonicity along soft coordinates is accounted for through molecular dynamics samplings, whereas stiff harmonic modes are treated at quantum vibronic level on effective PES that depend on the instantaneous position of the soft DoFs.

*FCclasses3* program has been strongly re‐structured, making it much more modular for helping its reading and modification. The main input style is completely renovated with a easy‐to‐follow keyword‐based structure. Special care was put to make the code easily usable in combination with different codes, input sources, and computational protocols. Therefore, the code accepts inputs in both Cartesian and normal coordinates basis (or mixed forms), we establish a transparent and well documented format for providing the auxiliary input data, like geometries, and energy and transition amplitude derivatives, and we provide interface tools that automatically generate such data from many widespread quantum chemistry codes. Graphical tools for helping visualization and assignment of the results are also furnished. Finally, the distribution package includes a large number of examples designed to test the installation and help the user learn how to apply the code.

The paper is organized as follows. In Section 2, we briefly review the main theoretical foundations for the different methodologies implemented in the code, including the definition of the harmonic models in Cartesian and curvilinear coordinates. For the sake of brevity, the actual equations (within TI or TD formulations) implemented in the codes are presented in the main text only for one‐photon spectroscopies, as an example, whereas all equations and new derivations are reported in the Supporting Information (S1). Section 3 details the code usage and the new distributed pre‐ and post‐processing tools. Section 4 is then devoted to some test applications, aiming at showing both the simple workflow to deal with the calculation of different properties and some key features to cope with scenarios where the harmonic approximations is challenged. Finally, Section 5 reports concluding remarks discussing possible perspectives for further development within the code.

## 2 IMPLEMENTED METHODS

### 2.1 Harmonic PES models and normal mode analysis

In *FCclasses3* both initial and final states are described by quadratic PES, that is, retaining up to the second term in the Taylor expansion around the reference geometry. The corresponding normal modes are adopted as a set of coordinates for each state to pose the vibronic problem. Normal coordinates are generally different for each electronic state, and they are related through the Duschinsky relation,^57^ which in the form used in *FCclasses3* reads:

(1) $$Q_{1}=JQ_{2}+K$$

where *Q*_1_ and *Q*_2_ refer to the normal modes at the initial and final states, *J* is the so‐called Duschinsky matrix and *K* are the normal mode displacements.

*FCclasses3* can adopt both adiabatic (A) and vertical (V) approaches to build up the harmonic PES. In fact, in most common vibronic approaches the initial‐state PES is always quadratically expanded around its own equilibrium geometry (it is however noteworthy that *FCclasses3* provides some generalization, described below). In contrast, for the final state, the expansion is usually performed around either the equilibrium structure of that state (A) or the equilibrium structure of the initial state (V), that is, the so‐called Franck‐Condon point. In previous works,^58^ we have designated such strategies as Adiabatic Hessian (AH) and Vertical Hessian (VH) models, respectively. Once normal modes are computed for the initial and final states, the Duschinsky matrix is evaluated as,

(2) $$J=L_{1}^{-1}L_{2}$$

The calculation of the normal mode displacements depends on the model adopted. For A models, it simply corresponds to the difference between the reference equilibrium geometries:

(3) $$K=L_{1}^{-1}\left(\xi_{1}^{\mathrm{eq}}-\xi_{2}^{\mathrm{eq}}\right)=L_{1}^{-1}\Delta \xi^{\mathrm{eq}}$$

where *ξ* is the set of coordinates adopted, either Cartesian or valence internal ones.

In the case of V models, the shift is evaluated finding the minimum of the quadratic final state PES, using both the gradient and the Hessian. It should be noted that, when V models are applied, normal modes for the final state are computed at a nonstationary point of the PES, so that in Cartesian coordinates it is not possible to rigorously separate rotations and vibrations.^59^ Indeed, in this case, the use of curvilinear set of coordinates is required, such as valence internal coordinates. In *FCclasses3*, the minimum over the final state potential is first obtained in valence internal coordinates:

(4) $$\Delta s=–{H_{s}}^{–1}g_{s}$$

where *H*_s_ and *g*_s_ are the Hessian and gradient for the final state in terms of valence internal coordinates. This displacement is then transformed to normal modes as indicated in Equation (3).

Simplified versions of adiabatic and vertical models can be generated. The most popular approximation imposes that all states share the same energy Hessian, leading to models that have many names in literature such as independent mode displaced harmonic oscillator (IMDHO) model^60^ or linear coupling model (LCM)^11^ and that we proposed to refer to as adiabatic shift (AS) and vertical gradient (VG) models so to make apparent the family of models they belong to (A or V).^58^ Intermediate‐quality models can be obtained keeping the same normal mode coordinates for both the initial and final states but updating the frequencies of the final state with the diagonal elements of the final state Hessian, leading to ASF and VGF models (where F stands for frequencies). A critical assessment of the relative quality of these models can be found in Ref. 58.

**FIGURE 1:**
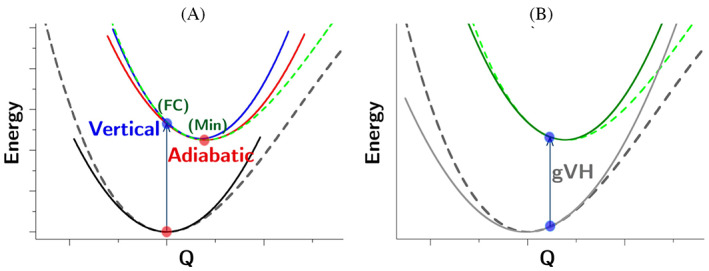
Schematic representation of (A) adiabatic Hessian and vertical Hessian models and (B) generalized vertical Hessian models (gVH).

V and A models are schematized in Figure 1 (panel A), highlighting the reference points where the quadratic PES are expanded. The VH model better describes the final‐state PES around of the initial‐state minimum, that is, the region more relevant for the absorption maximum. At the same time, it adds some uncertainty to the energy difference between the minima of both states (adiabatic energy). On the contrary, AH approach gives a better description of the low‐part of the spectrum, the so called, 0–0 region, but may introduce inaccuracies in the region of the absorption maximum. Marked differences between AH and VH predictions suggest the existence of relevant anharmonic effects.^58^

The same philosophy behind the VH model can be generalized, taking an arbitrary reference geometry. Such a generalized vertical Hessian model (gVH) is schematized in Figure 1 (panel B). This model can be helpful for specific applications, for instance when extracting geometries from an unconstrained MD sampling of the chromophore in an explicit solvent model.^56^ Since the reference point corresponds to a nonstationary point, gVH can only be applied adopting curvilinear coordinates.

The use of valence internal coordinates requires first selecting a nonredundant set of such elements, which is not unique. The coordinate space is then characterized by the Wilson B matrix, which relates valence internal, s, with Cartesian coordinates (*x*), *s* = Bx, and *G* = BM^−1^B (where *M* is a diagonal matrix with the atomic masses). In *FCclasses3*, a nonredundant set of coordinates is obtained from a redundant set that includes as all bonds, valence angles and dihedral angles arising from the connectivity pattern, leading to a set of delocalized internal coordinates. The coefficients of the combination can be obtained by single value decomposition of the Wilson B matrix^49^, ^61^ or from the non‐zero eigenvalues of the G matrix.^62^, ^63^

The Hessian in curvilinear valence internal coordinates is obtained from that in Cartesian coordinates with the following expression:

(5) $$H_{x}=B^{t}H_{s}B+g_{s}^{t}\beta$$

where $g_{s}^{t}$ is the gradient in internal coordinates (thus vanishes at stationary points) and *β* is a rank‐3 array whose elements correspond to the derivatives of the B matrix with respect to Cartesian coordinates. Although the latter can be evaluated analytically, *FCclasses3* implements numerical derivatives which revealed faster to compile and very stable and precise.^48^

As mentioned above, internal coordinates are more convenient than Cartesian to apply the AH model when large displacements are found for curvilinear modes between the initial and final state minima.^47^ Moreover, they are the only rigorous choice for VH model.^48^ However, it should be noted that they also find some limitations. Namely, the G matrix actually changes with the coordinates, which leads to additional nonharmonic terms in the Hamiltonian.^64^ Such terms are ignored within *FCclasses3*, assuming that the G does not change drastically from the initial to final state minima. One way to analyze the significance of such terms is comparing the lineshape obtained with the VH model evaluating the G matrix at either the initial state geometry (default choice) or the extrapolated minimum of the final state.^48^ This test can be performed within the code with a simple keyword in input.

#### 2.1.1 Reduced‐dimensionality models in curvilinear internal coordinates

Valence internal coordinates further provide a suitable framework for projecting out some selected DoFs from the coordinate space used for vibronic calculations at a harmonic level. This strategy can be particularly useful for flexible coordinates related to anharmonic regions of the PES. It is indeed the basis of hybrid methods, which retain the harmonic description for the stiff modes while treating the anharmonic ones with more appropriate methodologies.^56^, ^65^, ^66^

The projection to remove a curvilinear element has been already described in the literature,^67^ and it can be formulated as follows^56^:

(6) $$P=1-G\frac{gg^{t}}{{\left|g\right|}^{2}}=1-\frac{ss^{t}}{{\left|s\right|}^{2}}G^{-1}$$

where both the expressions both covariant, such as the gradient, *g*, and contravariant objects, such as an internal coordinate, *s*, are given. Note also that the calculation of the modulus generally includes the metric tensor, that is, |s|^2^ = s^*t*^G^−1^ s and |g|^2^=g^*t*^Gg.

Several coordinates can be removed by iteratively applying and updating the projector, as we recently proposed.^56^ The actual protocol implemented in *FCclasses3* is summarized in Figure S1.

### 2.2 Supported spectroscopies and photophysical properties

*FCclasses3* implements many different spectroscopies, namely OPA, EMI, ECD, CPL, MCD, TPA, TPCD and RR, and the computation of nonradiative rates driven by nonadiabatic couplings, IC, or any scalar coupling expanded up to the first‐order in terms of the nuclear coordinates. In order to avoid a too long manuscript we give most of the details and derivations in the Supporting Information. Here in the manuscript, we only briefly discuss one‐photon spectroscopies (OPA, EMI, ECD, and CPL) which can be described with common equations and allows us to introduce both TI and TD approaches.

The calculation of the spectrum, *S*(*ω*), which corresponds to the quantities experimentally recorded for each spectroscopy (molar absorptivity *ε* for OPA, its anisotropy Δ*ε* for ECD, number of emitted photons per unit of time *I*_emi_ for EMI, …) can be formulated as,

(7) $$S\left(\omega \right)=C\omega^{n}L\left(\omega \right)$$

where *L*(*ω*) is usually referred as the lineshape and *n* is an integer whose value also depends on the type of spectroscopy. Concretely, *n* = 1 for absorption processes (OPA, ECD) and *n* = 3 for emission (EMI, CPL). *C* is a constant that depends on the type of spectroscopy. As an example for OPA, when all variables are described in atomic units, *C* ≈ 703 leads to intensity as *ε*(M^−1^ cm^−1^).^68^

The expression for the lineshape at temperature *T* within a TI formalism is given by the following sum‐over‐states,

(8) $$L\left(\omega \right)=\sum_{v_{i}}\sum_{v_{f}}\rho_{v_{i}}\left(T\right)|\langle v_{i}|\mu_{\text{if}}{\left|v_{f}\rangle \right|}^{2}\delta \left(\omega_{f}-\omega_{i}\pm \left(\Delta E/\hbar -\omega \right)\right)$$

where Δ*E* is the adiabatic energy difference between the two PES in their minima, $\rho_{v_{i}}=e^{-\beta \hbar \omega_{i}}/Z_{\mathrm{vib}}$ (*β* = 1/*k*_*B*_ *T*, *k*_*B*_ being the Boltzmann constant), *Z*_vib_ is the vibrational partition function in the initial state and *δ* is the Dirac delta function which selects the energy corresponding to the transition. The ± sign depends on the type of transition, being “+” for absorption and “−” for emission, and it arises from the resonance condition. In practice, this *δ* function is replaced by a convenient broadening profile, *g*(*ω*), with finite broadening. Normally, Gaussian or Lorentzian profiles are adopted; other choices can be sometimes useful, such as a combination of both (Voigt profile) or the use of exponential decay functions.^69^ *μ*_if_ is the electronic transition dipole. Note that for ECD/CPL, the square module of the transition dipole integral is replaced by the product of the electric and the imaginary part of the magnetic transition dipole integrals.^12^

The equivalent expression within the TD formulation is given by the following Fourier transform:

(9) $$L\left(\omega \right)=\frac{1}{2\pi }\int \mathrm{dt}\;e^{\mp \mathrm{it}\left(\Delta E/\hbar -\omega \right)}\chi \left(t,T\right)g^{\prime }\left(t\right)$$

where *g* ^*’*^(*t*) is a damping function related to the broadening of the spectrum. Gaussian, Lorentzian and Voigt broadening profiles are implemented in *FCclasses3* in combination with a TD calculation. *χ* is the transition dipole correlation function, which for OPA/EMI is given by,

(10) $$\chi \left(t,T\right)=Z_{\mathrm{vib}}^{-1}\mathrm{Tr}\left[\mu e^{-i{\hat{H}}_{f}\tau_{f}}\mu e^{-i{\hat{H}}_{i}\tau_{i}}\right]$$

where $\tau_{f}=t/\hbar$ and $\tau_{i}=-\mathrm{i\beta}-t/\hbar$, and ${\hat{H}}_{i}$ and ${\hat{H}}_{f}$ are the Hamiltonians for the initial and final states.

Analytical expressions are available for both the vibronic transition dipole integrals,^14^ ⟨v_*i*_|*μ*_*if*_|v_*f*_⟩, and the correlation function *χ*(*t*, *T*),^22^, ^23^, ^26^, ^42^, ^70^ when the harmonic approximation is adopted and the electronic transition dipoles are described with FC and HT terms.

Finally, in the case of emission, the code also computes the radiative rate constant from the integration over the emission spectrum,^29^

(11) $$k_{r}=\int_{0}^{\infty }\frac{4\omega^{3}}{3\hbar c^{3}}L_{\mathrm{emi}}\left(\omega \right)\mathrm{d\omega}$$

Whereas TD approaches are eigenstate‐free approaches based on the fast evaluation of analytical expressions for time‐correlation functions and their Fourier transform, TI approaches are based on the evaluation of the spectra and properties through an explicit sum over all the possible vibronic states. This is in principle a challenging problem, since their possible number increases so steeply with the dimension of the system that a brute‐force calculation is out of the current possibilities for classical computers for the typical energy range covered by a spectrum and medium‐size molecules. In fact such number is ∼10^20^ for coumarin 153 (102 normal modes) for an energy window of ∼ 1 eV.^19^ Such a challenge is so well recognized that vibronic computations were individuated as ideal test cases for showing quantum advantage in quantum computers.^71^ Different approaches have been proposed to deal with this issue. *FCclasses3* implements a pre‐screening methodology already adopted in the previous version of the code, here extended to deal with additional spectroscopies/properties. It is based on a partition of the possible transition in classes $C_{n}$ depending on *n*, the number of simultaneously‐excited modes in the final‐state. Briefly, $C_{1}$ and $C_{2}$ always contain a moderate number of relevant transitions and are computed up to very high excitations so to ensure full convergence. Afterward, $C_{1}$ and $C_{2}$ data (and also those of additional classes for spectra not at 0 Kelvin^33^ or with HT contributions^72^) are analyzed to pre‐select the relevant transitions (within a maximum number set by input) to converge the contribution of higher‐order classes $C_{n}$. Further details can be found in Refs. 19, 33, 72. For this reason *FCclasses3* also provides as output the contribution to the spectrum of each specific class: fundamental and overtones, combinations bands of two‐, three‐ modes, and so on.

## 3 RUNNING *FCCLASSES3*

In this section, we review some details about the usage of the code, including the structure of the input file and the description of the tools provided with the package. More details about the implementation, for example, regarding the Fourier Transform, are given in the Supporting Information S1.

### 3.1 Input and output files

The program options are controlled by an input text file structured in lines stating OPTION=VALUE assignments. The order of entries is flexible, and sensible defaults are given to many options to facilitate job preparation. An example input file is given for the first test case below (Listing 1).

Additionally, the user must also provide the information about the PESs (structure, energy, gradient and Hessian) of the initial (1) and final (2) states through the state files (STATE1_FILE, STATE2_FILE), and about the coupling term (in the case above, electronic transition dipole moments) through other additional files, specified in the input. They all follow an intuitive format designed explicitly for *FCclasses3* and described transparently in the manual to allow users to easily compose the needed data from any available source. In particular we also provide some tools (described below) to automatically generate these input files from the output files of different popular quantum‐chemistry programs. It is worthy to specify that for HT computations *FCclasses3* can accept derivatives computed at the equilibrium geometry of both the initial (HTi) and final (HTf) state.

*FCclasses3* prints out several output files. In the standard output the user finds details of the computations, the vibrational analysis (displacements, frequency changes, projection of the normal modes), the analysis of relevant data (e.g., the transition dipole derivatives) and the computation of the total intensities for the different spectroscopies. The latter quantities are evaluated from analytical sum rules derived and presented for the different spectroscopies in the past (for OPA/EMI in Ref. 72, for ECD in Ref. 73, 74, for TPA/TPCD in Ref. 12, for MCD in Ref. 13, for RR in Ref. 75). They are particularly relevant for TI calculations since the convergence of the calculation is monitored in the standard output comparing the numerical sum of the transitions selected with the pre‐screening techniques with the analytical sum. Beyond the standard output *FCclasses3* prints out several additional output files where ready‐to‐plot formats for the convoluted spectra are found together with additional information (for TI calculations stick transitions and their assignments, for TD calculations correlation functions) and data for possible analysis of user's post‐processing of the calculations (like the Duschinsky matrix, or the transition polarizability tensor for RR calculations).

### 3.2 Pre‐ and post‐processing tools

Some tools aimed to facilitate job preparation and the analysis of the results are provided along with *FCclasses3*. Pre‐processing tools include two codes to generate the state files with information about the PES (gen_fcc_state) and coupling terms including transition dipoles and nonadiabatic coupling vectors (gen_fcc_dipfile) from output files of several quantum‐chemistry codes. Supported (or partially supported) formats include Gaussian (log and fchk), Psi4, Turbomole, Gamess, QChem, (Open)Molcas, Cfour, and Orca. State files can also be generated from molecular mechanics (MM) force fields evaluated with Gromacs. With these tools, we aim at fostering the interface of *FCclasses3* with different quantum chemistry codes. In this direction, the source code of such tools is open for contributions from the community, and to that end it is distributed also separately in a public git repository.^76^

Computed spectra are typically convoluted with a phenomenological linewidth meant to simulate the effect of different factors like the finite lifetime of the excited state and/or the solvent inhomogenoeus broadening. Such linewidth can be applied once the transitions (for TI) or correlation function (TD) have been computed. The code writes this information to files during execution, allowing to apply different broadening functions post‐processing the data without the need to re‐run the program. The tools reconvolute_TD and reconvolute_TI allow setting a new profile, among Gaussian, Lorentzian, decaying exponential (left or right) or a combination of them (like a Voigt profile), and a new width, indicated in all cases by a common parameter: the half‐width at half maximum (HWHM).

In the case of TI calculations, *FCclasses3* writes information about the most relevant transitions to a file. A graphical user interface (GUI) (fcc_analyzer_PyQt5.py), written in python and relying on PyQt5 libraries, is also provided to facilitate the inspection of these results. The interface allows the assignment of stick transitions in terms of the initial and final vibrational state (with labels that indicate the modes involved and the excited quanta), the selection of a given progression, the interactive manipulation of the width and the direct comparison with reference data (e.g., experimental spectrum). See an example in the first test case below.

For RR simulations, the output provides the stick transitions (along the Raman shift axis), which can be convoluted with Gaussian or Lorentzian curves with the tool convolute_RR. Moreover, an additional GUI, based on the PyQt5 framework, is also provided to assist the inspection of RR spectra, fcc_RRinspector_PyQt5.py. It allows the interactive change of the incident frequency to quickly visualize the effect of this parameter on the RR spectra (an animation showing the use of this tool for one system treated in the work is included in the SI).

## 4 RESULTS

Many of the new features of the code with respect to the distributed version *FCclasses* ^35^ have been applied in the recent years by our group and are now made available to the community. Among them we mention different spectroscopies, such as RR,^77^ TPA and TPCD,^12^ MCD^13^ or absorption with vibrational pre‐excitation,^34^ and nonradiative transitions, either triggered by nonadiabatic couplings^78^ or by any coupling independent of the coordinates (in ref. 79 we considered for example the intersystem crossing). However, specifically for this work, we implemented the new possibility to run TD calculations of TPA, TPCD, VIPER, and RR spectroscopies. In this section, we first show test computations for anthracene, a typical system in which harmonic approximation works smoothly. For this system, we systematically compute different properties, including OPA, EMI, TPA, RR, and IC rate, showing how these computations can all be run with simple changes in the input file, and how the GUI helps to analyze the results. These examples also allow us to show at work the new implementations of TPA and RR within the TD formulation. Afterward we show the new capabilities of the code in internal coordinates by computing the absorption and emission spectra for systems with few flexible coordinates (torsions), which represents one of the main challenges in the field that can be almost routinely approached with our code.

### 4.1 An example of a rigid system: Anthracene

In this section, we focus on anthracene, a paradigmatic case where the harmonic approximation delivers an extremely good framework to simulate the electronic spectroscopy.^58^ We use this example to illustrate the new easy‐to‐use workflow, from input preparation for the simulation of different properties, to the analysis of the stick transitions. As indicated above, the use of the pre‐processing tools to extract the relevant data from the output of electronic structure program along with the generation of template inputs, allows the user to quickly set up all required files to run a calculation. All calculations in this section were carried out using DFT and TDDFT in the gas phase, adopting the B3LYP functional in combination with the 6‐31G(d) basis set. Gaussian16^38^ was used to perform all electronic structure calculations, except for the computation of the two‐photon transition tensors, which were computed with Dalton 2016.^80^

We start simulating the OPA spectrum. The required input to carry out the simulation of the spectrum at 300 K is shown in Listing 1. Although almost all keywords adopt sensible defaults, we make explicit the most relevant settings (all input files required to run the examples in this section are given in the Supporting Information). The input specifies the harmonic model, AH, and the approximation to expand the electronic transition dipole, FC. Moreover, we indicate the type of broadening function to be applied (Gaussian) and its width (HWHM = 0.01 eV). The TD method is selected to compute the spectrum, which is very convenient to get a converged spectrum at 300 K within a reasonable CPU time. Note that the parameters controlling the sampling of the correlation function are set automatically from the resolution (by default 1/10 of the HWHM) and the frequency range (also selected automatically) in which the spectrum is printed out (see SI, Section 3.4). The normal mode analysis is carried out by the code (alternatively, the vibrational or vibronic analysis could be read from external files), and Cartesian coordinates are used to describe normal modes. Finally, the files containing the information to build the harmonic model PESs of the initial and final states are given in anthracene_S0‐f.fcc and anthracene_S1‐f.fcc, while transition dipole is given in eldip_anthracene.

LISTING 1 **Example input file structure for the calculation of a one photon absorption spectrum of anthracene at 300 K. Text after semicolons is ignored.**

$$$

PROPERTY = OPA; OPA/EMI/ECD/CPL/RR/TPA/TPCD/MCD/IC/NRSC

MODEL = AH; AS/ASF/AH/VG/VGF/VH

DIPOLE = FC; FC/HTi/HTf

TEMP = 300.00; (temperature in K)

BROADFUN = GAU; GAU/LOR/VOI

HWHM = 0.01; (broadening width in eV)

METHOD = TD; TI/TD

;VIBRATIONAL ANALYSIS

NORMALMODES = COMPUTE; COMPUTE/READ/IMPLICIT

COORDS = CARTESIAN; CARTESIAN/INTERNAL

;INPUT DATA FILES

STATE1_FILE = anthracene_S0‐f.fcc

STATE2_FILE = anthracene_S1‐f.fcc

ELDIP_FILE = eldip_anthracene

**FIGURE 2:**
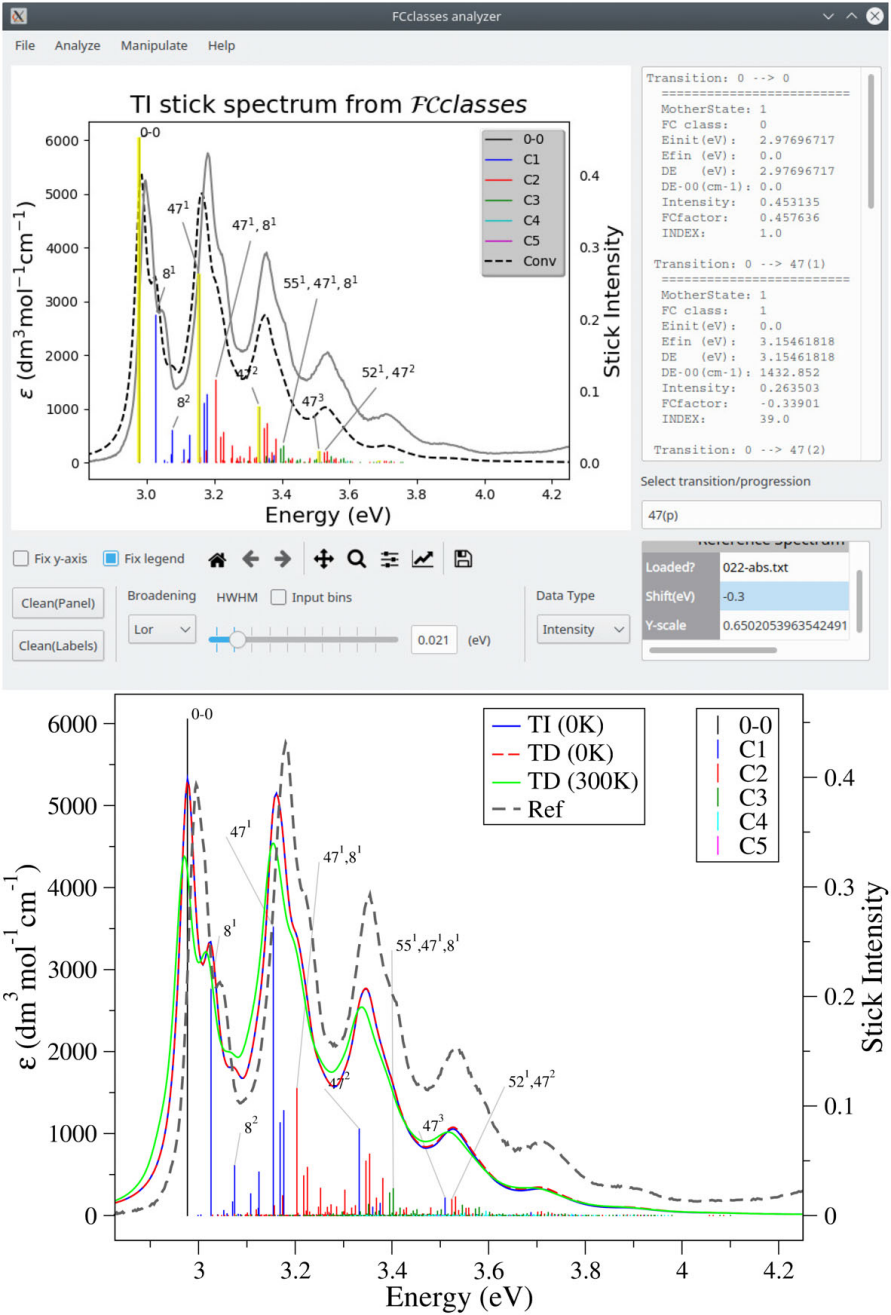
(Top) Screenshot of the GUI tool to analyze TI results showing the analysis of the TI absorption spectra of anthracene. It includes the experimental spectrum (shifted by −0.30 eV). For the convolution we used a Lorentzian function with HWHM = 0.02 eV. (Bottom) The same plot exported to xmgrace, where the TD spectra (at 0 and 300 K) have been added, computed with the same convolution scheme.

The above input leads to the absorption spectrum in ∼3 s on a Xeon processor. The TD method takes the same time also for a calculation at 0 K. The calculation with the TI method requires ∼11 s at 0 K (with the defaults settings) reaching a recovery of the total intensity >99%, while the calculation at 300 K, although feasible in principle, would require a much larger amount of time. The right panel of Figure 2 reports these spectra (all convoluted with a Lorentzian having a HWHM = 0.01 eV) showing that TI and TD computations deliver identical results at 0 K, as it should be. Moreover, application of the TD method at 300 K allows for an efficient inclusion of thermal effects, delivering a spectrum, where the relative intensity of the peaks follows the experimental trend. TI calculations provide information about the nature of the most relevant individual stick transitions, which is written to the file ASSIGNMENT.DAT during the calculations. The post‐processing tool fcc_analyzer_PyQt5.py can be used to more easily visualize all such information. In the top panel of Figure 2, we show a screenshot of this GUI analyzing the TI calculation for anthracene. The tool allows to overlap a reference spectrum (in this case the experiment in cyclohexane at room temperature), which can be shifted and scaled, and interactively apply different convolution schemes to the computed one. This helps us to determine that, in this case, a Lorentzian convolution with HWHM of 0.02 eV leads to the best agreement between the 0 K spectra and the experiment (at room temperature). Most of all, the tool facilitates the assignment of the different stick transitions and the main progressions (they can in any case be made manually by the reader inspecting the file ASSIGNMENT.DAT). In the screenshot, the main progression (for mode number 47: CC stretching) is highlighted, while some transitions are identified with a label *n* ^*y*^ specifying the vibrational mode of the final state that is excited *n* and its quantum number *y*. It is noteworthy that, although for high temperatures the TD algorithm get fully converged spectra in a much more efficient way than TI one, TI computations can still be used to identify the most relevant stick transitions and the GUI is able to provide their assignments. In order to facilitate the editing by the user, the plot, including labels, can be exported to xmgrace format. In bottom panel of Figure 2 actually show the plot in such a format.

The emission spectrum can be computed with a very similar input, setting PROPERTY = EMI and exchanging the data for the initial and final states. In addition to the emission spectrum, which for anthracene is nearly mirror symmetric with respect to absorption (see Figure S2), the calculation also provides the corresponding radiative rate (*k*_*r*_) computed from Equation (11). For this transition, that is bright and correctly described at FC level, the rate is mainly dictated by the value of the transition dipole moment, and only slightly tuned by the vibronic shape. A value *k*_*r*_ = 1.9 × 10^7^ s^−1^ is obtained, which is close to previous simulations^29^ and experiments.^81^

We now turn to explore the TPA spectrum of anthracene. Both S_1_ (*B*_1*u*_) and S_2_ (*B*_2*u*_) are TPA forbidden by symmetry. However, an experimental TPA signal in the spectral window associated to these states has been reported for anthracene in solution and assigned to the *B*_2*u*_ state.^82^ Here, we simulate the TPA spectra for both S_1_ and S_2_ that arise from the HT contribution in the gas phase. The two‐photon transition tensor is computed by quadratic response DFT, as implemented in Dalton. Derivatives are computed by finite differences along each normal mode, by applying positive and negative displacements with a dimensionless shift of 0.02. Such displaced geometries were generated with a local script.^83^ In order to ensure that exactly the same normal modes are used, we first run a *FCclasses3* job that writes normal modes to files, which are subsequently read by the external script. Following this procedure, and taking into account that the tensor at the reference geometry is exactly zero, we lose the trace of the sign of the derivative. The sign, however, is not relevant within VG model and, for this reason, this is the model adopted to run the vibronic calculations. Energies, gradients, and Hessians are evaluated with the same functional and basis with Gaussian16. The input for *FCclasses3* is similar to that of OPA. We simply need to change the keyword for the PROPERTY to TPA, and update the relevant settings for the state files, temperature (300 K) or harmonic model (VG). The two‐photon transition tensor and its derivatives along normal modes are read from a file prepared from the finite differences calculation mentioned above.

**FIGURE 3:**
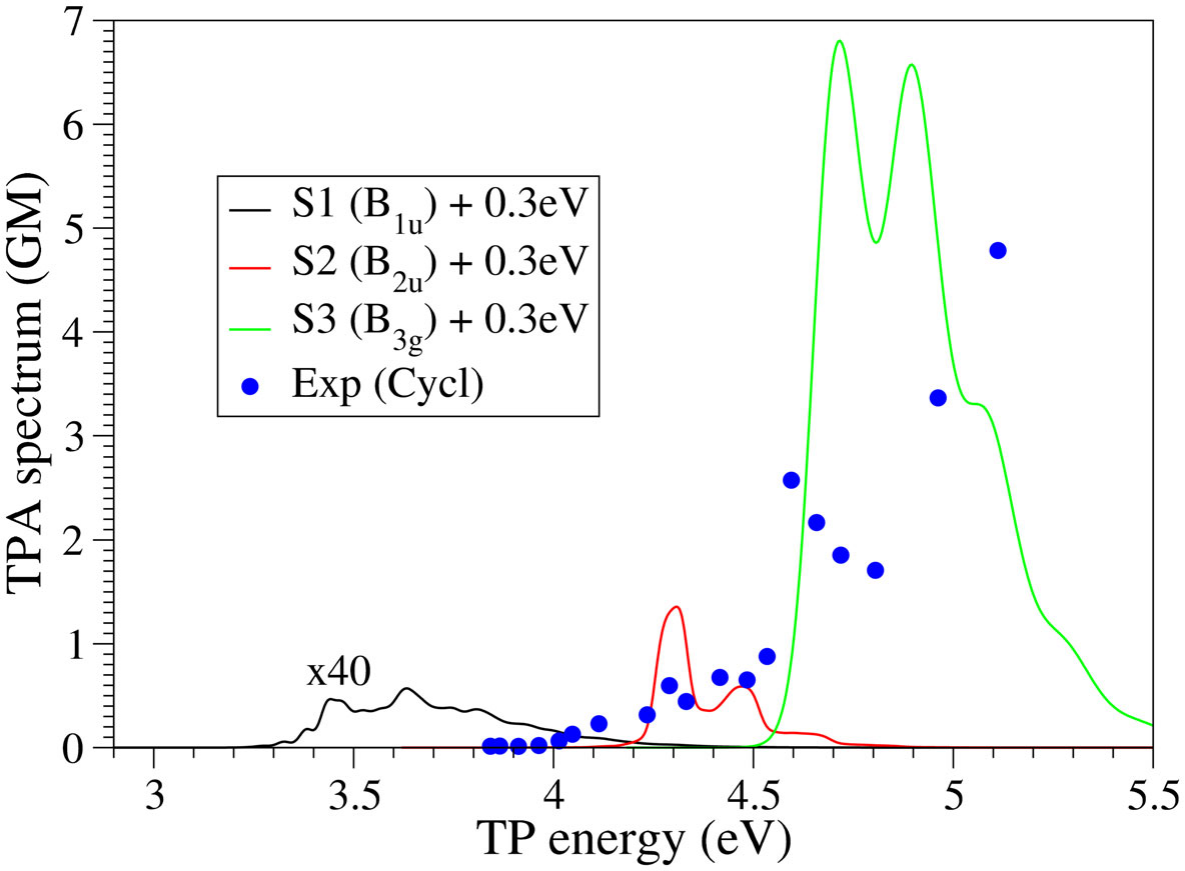
TPA spectrum of anthracene computed at 300 K with the VG model for S_1_, S_2_, and S_3_ states. S_1_, S_2_ (TPA forbidden) are computed with FC + HTi terms and applying a Gaussian broadening with HWHM = 0.02 eV. S_3_ (TPA bright) is computed including FC terms only and a Gaussian broadening with HWHM = 0.05 eV. The experimental TPA spectrum adapted from Ref. 84 is also included. Simulated spectra are blue‐shifted by 0.3 eV, consistently with the shift described for OPA in Figure 2. For better visualization, the S_1_ band is scaled by a factor of 40.

The simulated TPA spectra including the symmetry forbidden transitions to S_1_ (*B*_2*u*_) and S_2_ (*B*_3*u*_) and the TPA active S_3_ (*B*_1*g*_) are shown in Figure 3. HT effects are obviously required to reproduce the experimental observation of the symmetry forbidden bands, and the vibronic calculations included in *FCclasses3* perform quite well in this sense. In this case, the limited resolution of the experimental measurements, and limited accuracy of the TD‐DFT calculation, which does not account for potential multireference character,^84^ does not allow a careful assessment of the simulated lineshape. Still, our results provide a correct semi‐quantitative picture of the spectrum. Namely, the S2 (*B*_3*u*_) is much more intense than the S_1_ band, which is consistent with the experimental evidences, although the ratio between both bands seems to be overestimated.^85^ The TPA active transition leads to a more intense band, also in agreement with the experiment. Note that, for the latter, only FC terms are included in the calculation as it is a TPA bright transition. Overall, taking into consideration the shift that was already required for the OPA spectrum (0.3 eV), the general reproduction of TPA spectrum is not far from the experiments, and actually supports the assignments of the main bands provided in Ref. 85.

We continue the spectroscopic analysis of anthracene by simulating its RR spectrum (at 0 K). *FCclasses3* computed the RR spectrum as a two‐dimensional (2D) signal function of the excitation frequency and the Raman shift. Again, the calculation only requires slight changes to the input shown in Listing 1. Concretely, we need to set PROPERTY = RR, and update the files with the state files and electronic dipole data of the relevant root for this new property (we selected the brightest state S5, see below). Moreover, in this case, we adopt the VH model (using internal coordinates), which avoids the sometimes problematic optimization of the excited states. The RR calculation provides the sticks corresponding to fundamental, overtones of ground state normal modes and combinations bands (involving two modes). Their intensity depend on the incident frequency and on the damping function related to the lifetime of the excited state, *γ*_*k*_ (see Equation S15 in the Supporting Information). The simulation delivers the 2D RR spectrum at several incident frequencies (with common settings >1000), spanning a typical range of 25,000 cm^−1^ (3 eV). A convoluted spectrum (with respect to the Raman shift) for one specific incident frequency can be computed with the tool convolute_RR.

The experimental spectrum in different solvents recorded using an excitation frequency in the UV region has been recently reported including also a detailed computational analysis.^86^ Here, we will compare our gas phase results with those recorded in a nonpolar solvent, cyclohexane. The excitation frequency used in the experiments is nearly resonant with the peak of the brightest transition. According to our calculation in the gas phase, this state corresponds with S5 (*B*_2*u*_), which has a large oscillator strength (*f* = 1.9). We first simulate the OPA spectrum for the S5 state with the VH model, which results in vibronic profile with a maximum at the 0–0 band, consistent with the experiment, although appreciably narrower (see Figure S3 in the SI). The simulation of the RR is carried out taking the energy of the maximum of the simulated spectrum (∼5.1 eV) as incident frequency. We adopt the VH model and FC + HTi approximation we and account for fundamentals and overtones with two quanta for each mode whereas for combinations of two modes (1 + 1 quanta) we selected the modes with stronger fundamentals. For the computations, we use a damping function *Γ* = 150 cm^−1^, following the simulations already reported for this system,^86^ and convolute the resulting sticks with a Lorentzian function with HWHM = 5 cm^−1^ (along the Raman shift). Both TI and TD approaches are adopted, which deliver practically the same results (TI convergence is >99%; see Figure S4), although the computational time required for the TI calculation (up to overtones) is one order of magnitude larger as compared with the TD calculation (3700 vs. 350 s with a Intel‐i9 processor). Moreover, the TD calculation provides results for more than 2000 incident frequencies, while only 21 are computed with TI. In the following, we show the results with the TD method only.

**FIGURE 4:**
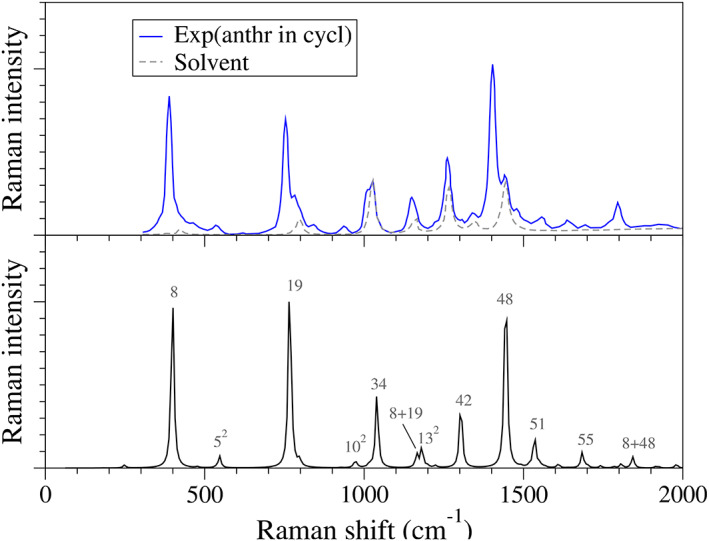
(Bottom) RR spectrum for anthracene with the incident frequency at the maximum of the bright UV transition (S5 in gas phase), computed with the VH model and FC|HTi dipole terms, and convoluting the stick transitions with a Lorentzian with HWHM = 5 cm cm^−1^. All fundamental and overtone bands are accounted for, as the combination between the most intense fundamentals (8, 19, and 42). The labels over the simulated spectrum indicate the final vibrational state of the transition. (Top) Experimental spectrum of anthracene (0.1 mM) in cyclohexane from Ref. 86.

The simulated spectrum at *ω*_*I*_ = 41,129 cm^−1^ (5.099 eV) is shown in Figure 4, including the comparison with the experimental one from Ref. 86. The agreement is excellent, which allows us to confidently assign the main vibrational peaks. Over the simulated spectrum we include the index corresponding to each normal mode involved in the fundamentals, overtones (indicated with a superscript of 2) and combination bands (indicated as the sum of involved modes). The description and symmetry of each mode is shown in Table S1. As expected by symmetry, almost all fundamentals correspond to *A*_*g*_ symmetry modes. The only exception is mode 55 (CC stretching), which belongs to *B*_3*g*_ and arises from the HT contribution. We also identify the overtones of modes 5 (*B*_2*g*_), 10 (*B*_1*g*_), and 13 (*B*_2*g*_), as well as the combination of the modes responsible for the most intense fundamentals, including 8 + 19 and 8 + 48. TD calculations provide the RR spectrum for a wide range of incident frequencies (37,000–67,000 cm^−1^). In order to easily explore the 2D data (RR intensity at different incident and scattered frequencies), the tool fcc_RRinspector_PyQt5 can be used, which allows us to interactively inspect the RR spectra at different incident frequencies (see movie in the Supporting Information S1).

Finally, we turn to explore the nonradiative internal conversion rates of anthracene from S_1_, triggered by the nonadiabatic coupling with the ground state. The nonadiabatic coupling vector is computed at TDDFT level as implemented in Gaussian16. The input is very similar to that for emission, but setting PROPERTY = IC and specifying, instead of the ELDIP_FILE, the NAC_FILE, which contains the nonadiabatic coupling matrix elements in terms of Cartesian coordinates.

**FIGURE 5:**
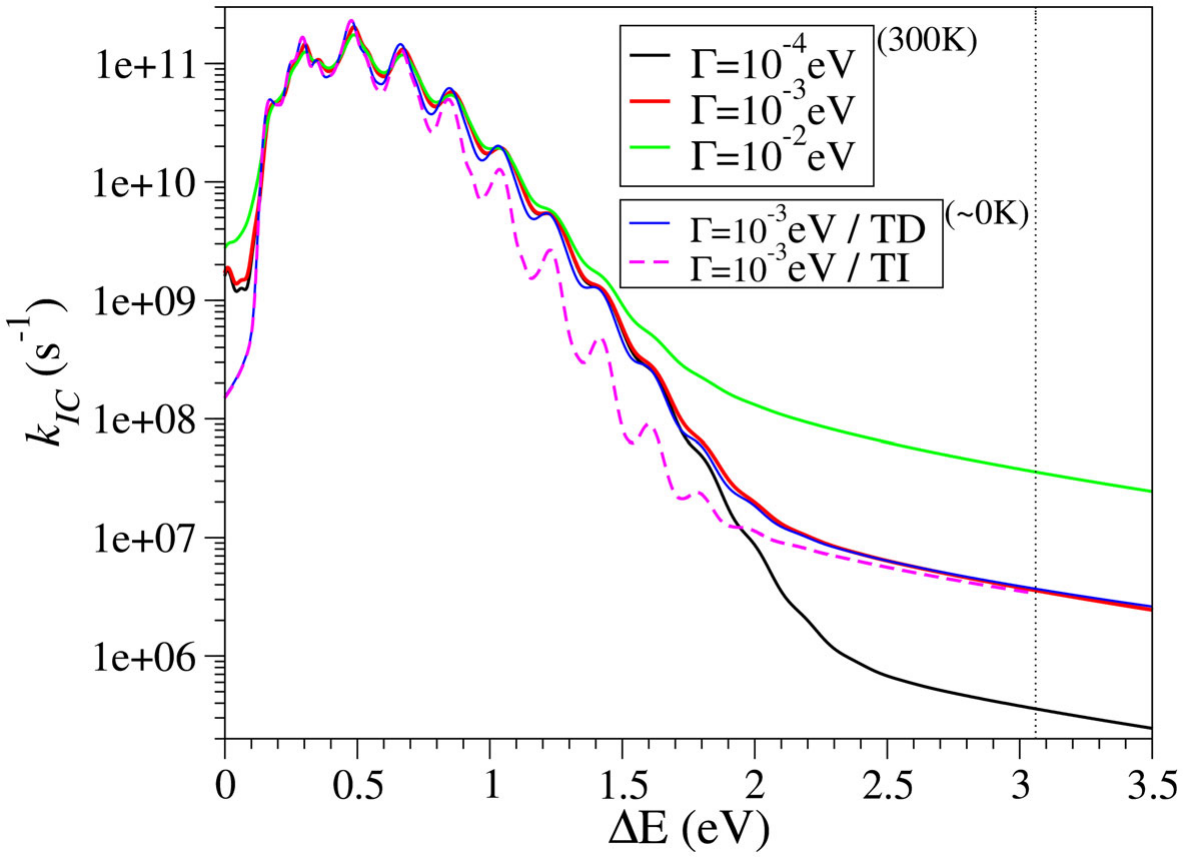
Dependence of the IC rate with the adiabatic energy for anthracene. The calculations are carried out with the AH model in Cartesian coordinates. A Voigt profile is used, with a Gaussian component wit HWHM = 0.02 eV and different settings for the Lorentzian component: from Γ(≡HWHM) from 10^−4^ to 10^−2^ eV. Simulations are carried out both at 300 K and at (nearly) 0 K. For the latter, the calculation are conducted with the TD and TI (convergence = 90%) methods. The vertical dotted line indicates the adiabatic energy of the system.

The profile for nonadiabatic rate vs the adiabatic energy obtained with the AH model is shown in Figure 5. We have applied a Voigt profile with different Lorentzian components. The subsequent convolutions are conducted with the reconvolute_TD and reconvolute_TI post‐processing tools (which allows to apply the Voigt profile also to TI calculations). The value of the rate constant shall be taken at the corresponding value of the adiabatic energy, indicated in the figure by a vertical line.^78^ As observed in this figure, for an adiabatic energies >1.5–2.0 eV, the value of the rate constant is mainly governed by the width of the selected Lorentzian broadening. Therefore, at the adiabatic energy of anthracene, 3.06 eV, is not possible to obtain a reliable value for *k*_*IC*_ if such broadening is introduced as a phenomenological parameter. It is also worthy to notice that for such large Δ*E* values significant effects of anharmonicities might be expected (see ref. 78). The plot also includes the results with the TI method at 0 K, with a convergence of 90%. Since the rate constant is typically taken at adiabatic energy values corresponding to the decaying wing of the function in Figure 5, far from the 0–0 transition, a region which usually challenges the convergence of the TI calculation, the adoption of this implementation is encouraged only for interpretative purposes (e.g., assignments of the role of specific progressions).

In this section, we have illustrated the versatility of the program and its ease of use. All calculations require a minimal input from the user, since many of the settings adopt sensible values, which can anyway be overridden with the corresponding keyword in input. The molecule adopted for this systematic analysis is rather rigid and represents a specially favorable case for the application of the harmonic approximation. In the next section, we will explore some systems where the harmonic approximation is not applicable in a straightforward way and discuss how can we approach such situations within *FCclasses3*.

### 4.2 Vibronic spectroscopy of flexible systems

**FIGURE 6:**
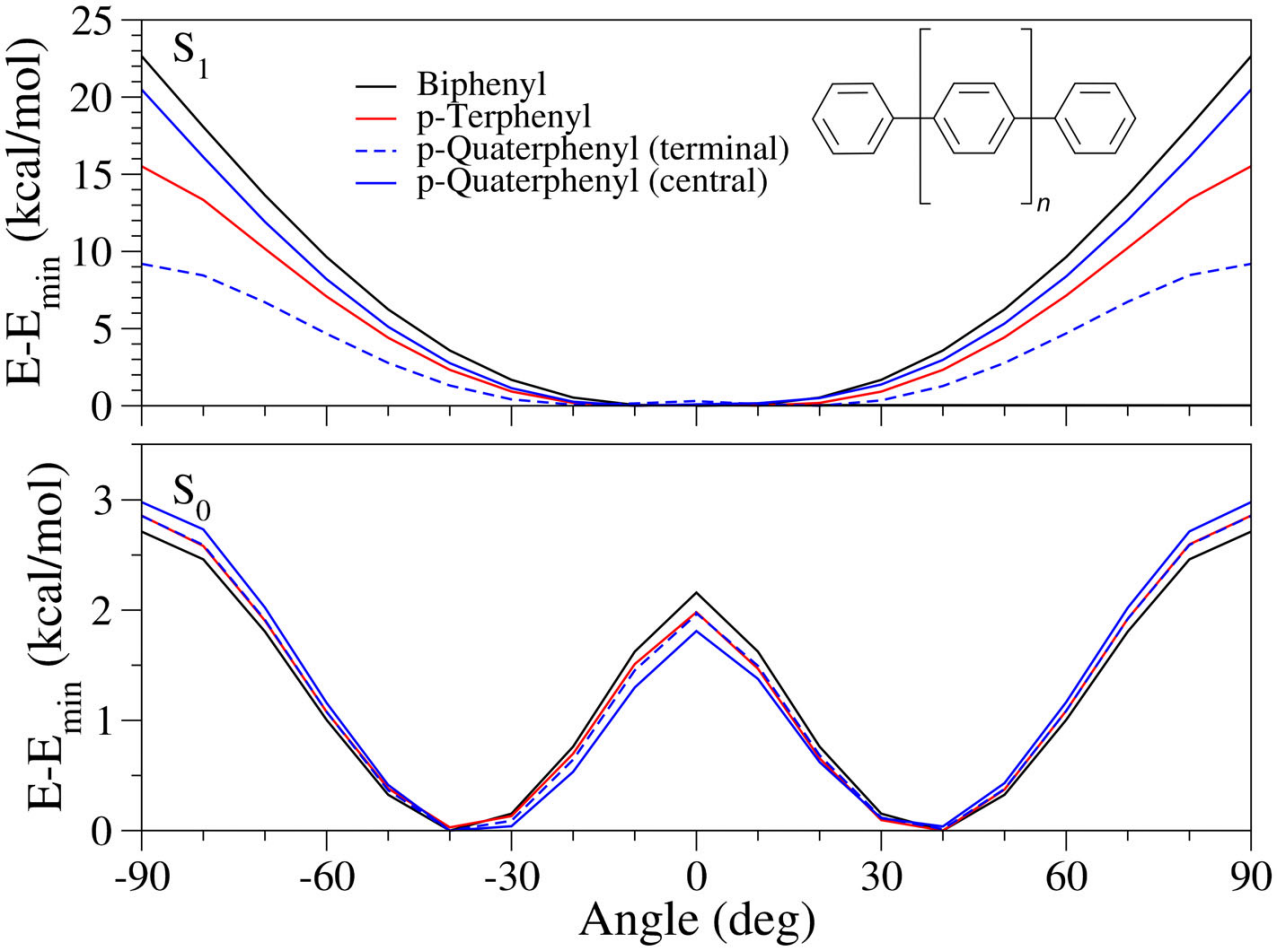
Relaxed potential energy curves corresponding to one flexible dihedral of the different polyphenyls under study at PBE0/6‐31G(d). Over the ground and excited (S_1_ which is the bright H → L transition). The profile for biphenyl is also included for comparison. Inset: Schematic representation of polyphenyl structure, where *n* = 1, 2, for p‐terphenyl and p‐quaterphenyl, respectively

We turn now to show some of the new features of the code, which can be especially useful to treat flexible systems. Concretely, we approach two polyphenyl molecules: p‐terphenyl and p‐quaterphenyl (see inset in Figure 6), whose experimental spectra are obtained from Ref. 87. The simplest molecule from this series, that is, biphenyl is excluded from this study as TDDFT faces some issues to describe low lying excited states, so that multireference approaches such as CASPT2^88^ or SAC‐CI^89^ would be required to deliver an accurate picture. The flexibility of the molecules is related to the inter‐ring torsions and put in crisis the deployment of the harmonic approximation. The interest of such systems in optoelectronics, for example, as building blocks for OLEDs, further motivates the accurate simulation of their optical properties.^90^ The energy, gradient and Hessian of the ground and bright excited states are evaluated with DFT and TDDFT at the equilibrium geometries of each state, so to be able to adopt both adiabatic and vertical models. The PBE0 functional along with the 6‐31G(d) basis set is adopted. The effect of the solvent (cyclohexane) has been accounted for with the IEFPCM model.^91^

In Figure 6, we show the energy profiles for all compounds along each inter‐ring torsion. They are very similar on S_0_, while they display some differences on S_1_. In S_0_, the curves are characteristic of single bonds, with the energy changes tuned by steric effects, comparable for all compounds. On S_1_, the increased double bond character leads to a profile with minima at *cis* and *trans* configurations and a maximum at 90°. The differences on S_1_ for each compound can be thus rationalized by the expected double bond character induced by the electronic transition. The bond order is actually maximum for biphenyl (included for comparison), which shows the highest energy barrier. For p‐terphenyl, the increment in bond order is spread over the two possible bonds equally, reducing the double bond character. For p‐quaterphenyl, finally, the transition is more localized over the central bond, so that the ES gets a double bond character similar to that of biphenyl. In contrast, the terminal bonds still show a significant single bond character, with much lower energy barriers.

The analysis of the energy profiles reveal some relevant features that are expected to impact the calculation of the electronic band shapes. First, the structural rearrangement along the torsion between S_0_ and S_1_ minima is rather significant, with a change of nearly 40°. This large curvilinear displacement makes unsuitable the use of linear Cartesian coordinates and this issue can be remediated by adopting curvilinear coordinates, such as valence internal coordinates.^47^ Second, the profiles, especially on S_0_, are clearly anharmonic which makes questionable the adoption of the harmonic approximation. Such features have a remarkable effect on the spectral shape; indeed, the differences in the shape of the S_0_ and S_1_ PES is responsible for a marked symmetry‐breaking between absorption and emission spectra.^92^

Such a situation can be approached with different strategies within *FCclasses3*, adopting in all cases curvilinear internal coordinates. In the following, we discuss the appropriateness of the different models available within the code.

**FIGURE 7:**
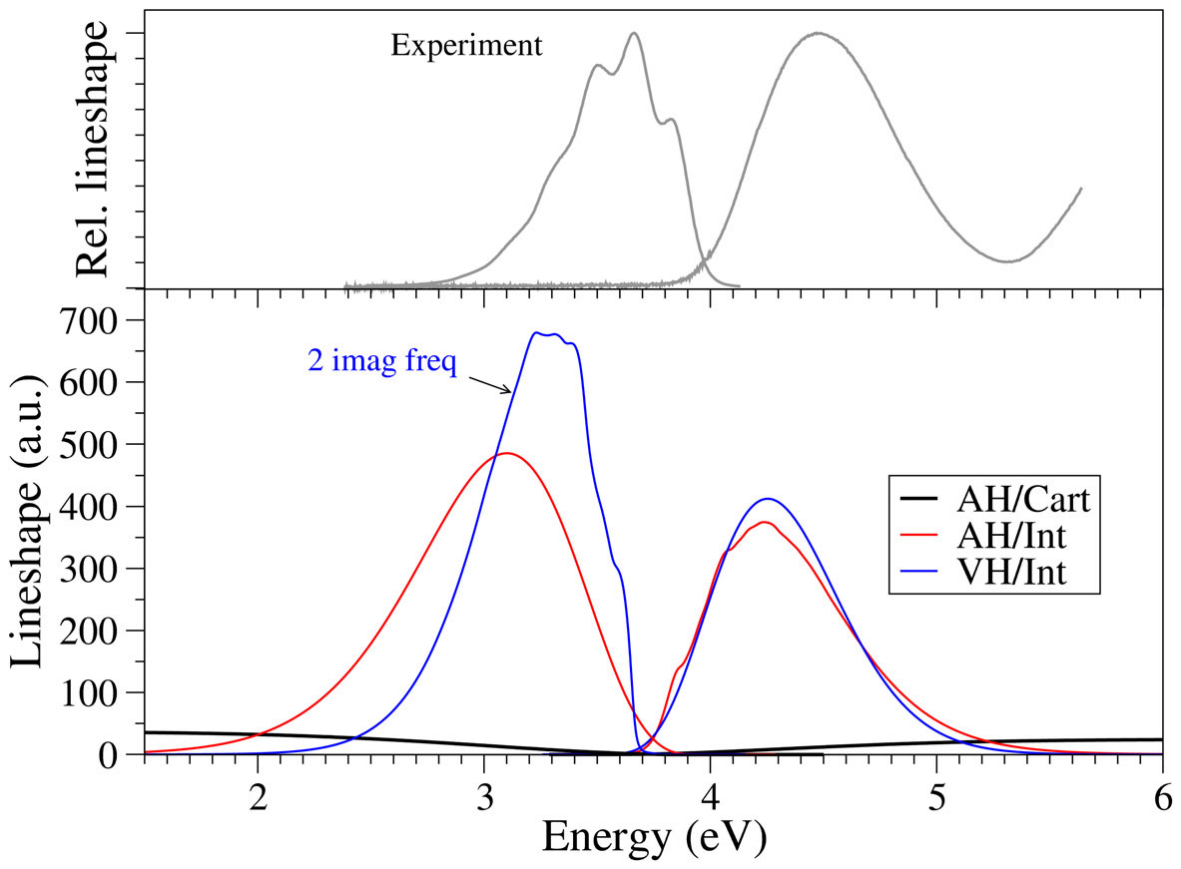
(Bottom) Absorption and emission spectra simulated at 300 K for p‐terphenyl adopting different harmonic models: AH (both in Cartesian and internal coordinates) and VH. In the simulation of the emission spectra with VH model an imaginary frequency arose, which was arbitrarily turned real. (Top) Experimental absorption and emission lineshape spectral, adapted from Ref. 87.

In Figure 7 we report absorption and emission spectra adopting both A and V models. The inadequacy of the AH model with Cartesian coordinates is made evident, as it provides an anomalously broad band shape both for absorption and emission. This outcome is a direct consequence of the inability of linear coordinates to describe curvilinear displacements, such as those along torsions.^47^ The use of curvilinear internal coordinates, instead, remediates such a limitation, leading to spectral shapes with a more reasonable width and much closer to the experiment. Regarding the shape, the absorption spectra retain some vibrational structure while emission are completely structureless, contrarily to what is observed in the experiment, where only emission spectra retain some vibrational features. Vertical models also perform reasonably well, and provide a good estimation of the absorption band shape. However, the simulation of emission faces the issues of anharmonicities at the final state (S_0_). Namely, two imaginary frequencies arise, related to the torsional modes, whose energy profiles in the ground state display a transition state at the minimum of the excited state. As a first crude approximation, a keyword in the input allows to simply turn the imaginary frequencies to real in order to complete the calculation. This strategy reveals appropriate in cases where the problematic modes do not play a significant role in the spectral shapes. The reliability of this approach can be tested by changing randomly the real frequencies to be used and checking if this affects the resulting spectrum. This check can be can be done straightforwardly from input, indicating the file with the values of the frequencies that will override the computed ones (while keeping the normal mode definition). Regarding the systems under investigation, the value used to replace the imaginary frequency has a large impact on the spectrum, (see Figure S5) signaling that for this specific case this simple strategy is not reliable.

A more refined treatment of the torsion requires going beyond the harmonic approximation, at least for this degree of freedom (DoF). This can be done within *FCclasses3* by exploiting the tools we developed to define reduced‐dimensionality models. They operate removing a set of internal coordinates defined as a given original internal coordinate (bond, angle, or dihedral) or a combination of them. Concretely, the torsion is the linear combination of all dihedrals involved, with equal coefficients. By removing this coordinate, the dimension of the internal space treated within *FCclasses3* within the harmonic approximation is reduced by one. The removed coordinate can then be treated independently to account for the actual potential energy profile more appropriately. A similar strategy was already adopted by some of us to study the absorption and emission spectra of dithiophene, which shows a torsion with a similar change between S_0_ and S_1._ ^55^ In that work, the contribution of the torsion was evaluated both variationally, that is, computing the anharmonic energy profile, fitting it and then diagonalizing the vibrational Hamiltonian on a basis of sines and cosines, and classically, that is, obtaining the distribution of vertical energies over the torsion according to a Boltzmann population. The final spectrum was computed by convoluting the profile corresponding to the torsion and the lineshape for the remaining DoFs treated quantum mechanically under the harmonic approximation. We can follow the same approach for p‐terphenyl and p‐quaterphenyl. Since the contribution of the torsion at room temperature was shown to be correctly described classically in Ref. 55, we here adopt the classical approach only, which has also the benefit that the computation can be in practice completed running only *FCclasses3*.

**FIGURE 8:**
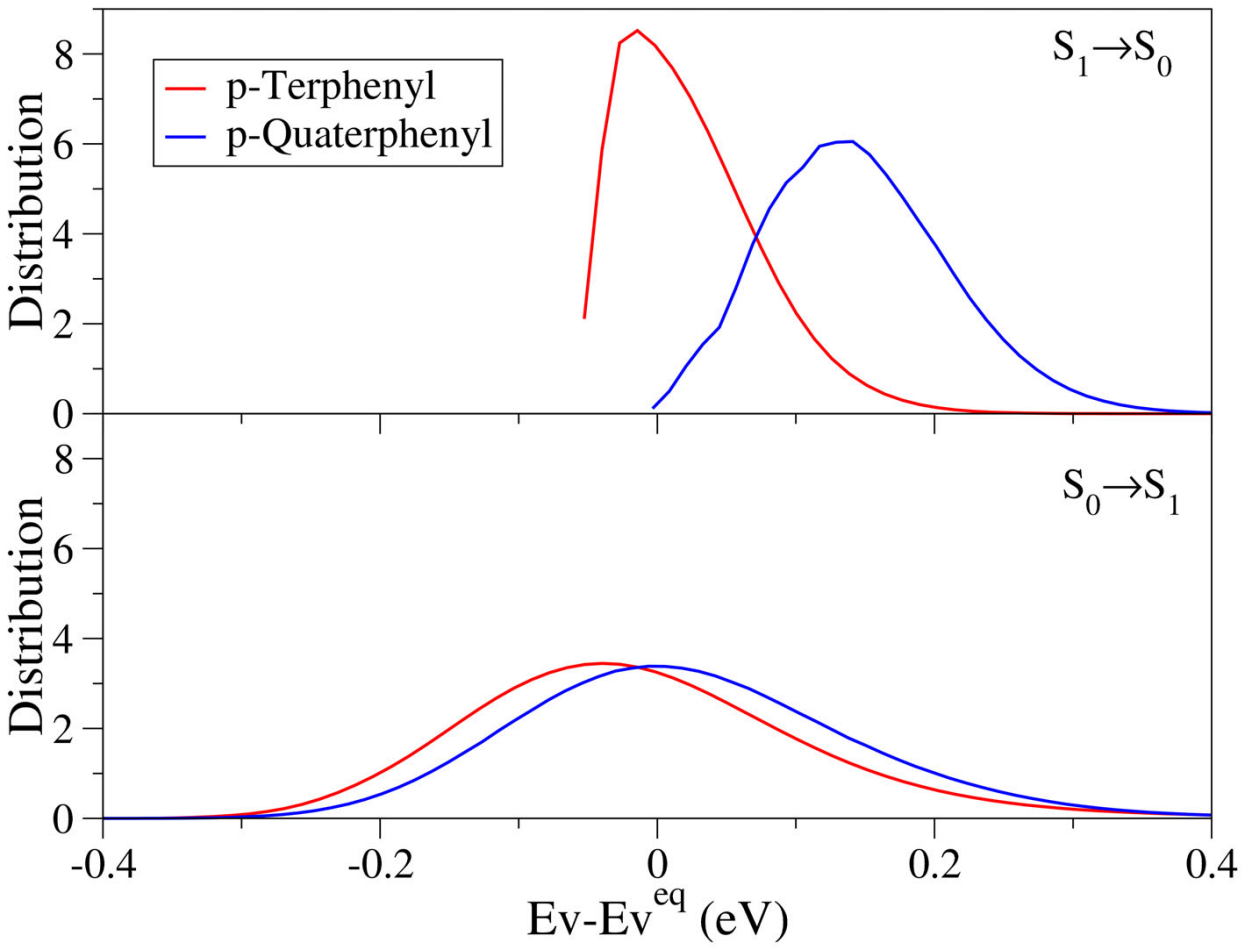
Distribution of transition energies at 300 K over the torsional degrees of freedom for p‐terphenyl and p‐quaterphenyl, both for *S*_1_ → *S*_0_ (top) and *S*_0_ → *S*_1_ (bottom).

The distribution that describes the torsion lineshape is computed by building a weighted histogram from the energy differences between the relaxed energy curves (Figure 6), and it is shown in Figure 8. We assume that the different torsional DoFs are independent within a given molecule, so that the total energy is computed by the sum of the individual (1D) relaxed scans. As shown in Figure S6, this is a good approximation for S_0_ and still remains reasonable for S_1_. A cubic interpolation has been used to increase the sampling along the curves, and the weights for the histogram have been computed as $\rho_{i}=e^{-E_{i}/k_{B}T}$, where *E*_*i*_ is the energy with respect to the bottom of the curve for the initial state.^93^ The actual contribution of the torsion to the spectral shape is modeled with a Gaussian with the same width (i.e., the same HWHM) as the distribution shown in the figure, which is then convoluted with the spectrum of the remaining harmonic DoFs. This strategy is particularly convenient as it allows us to simply use the broadening options in the input to include the effect of the torsion. Concretely, for p‐terphenyl the lineshape of the torsions have HWHM of 0.056 eV (for emission) and 0.133 eV (for absorption), while for p‐quaterphenyl we got HWHM of 0.078 eV (for emission) and 0.136 eV (for absorption).

**FIGURE 9:**
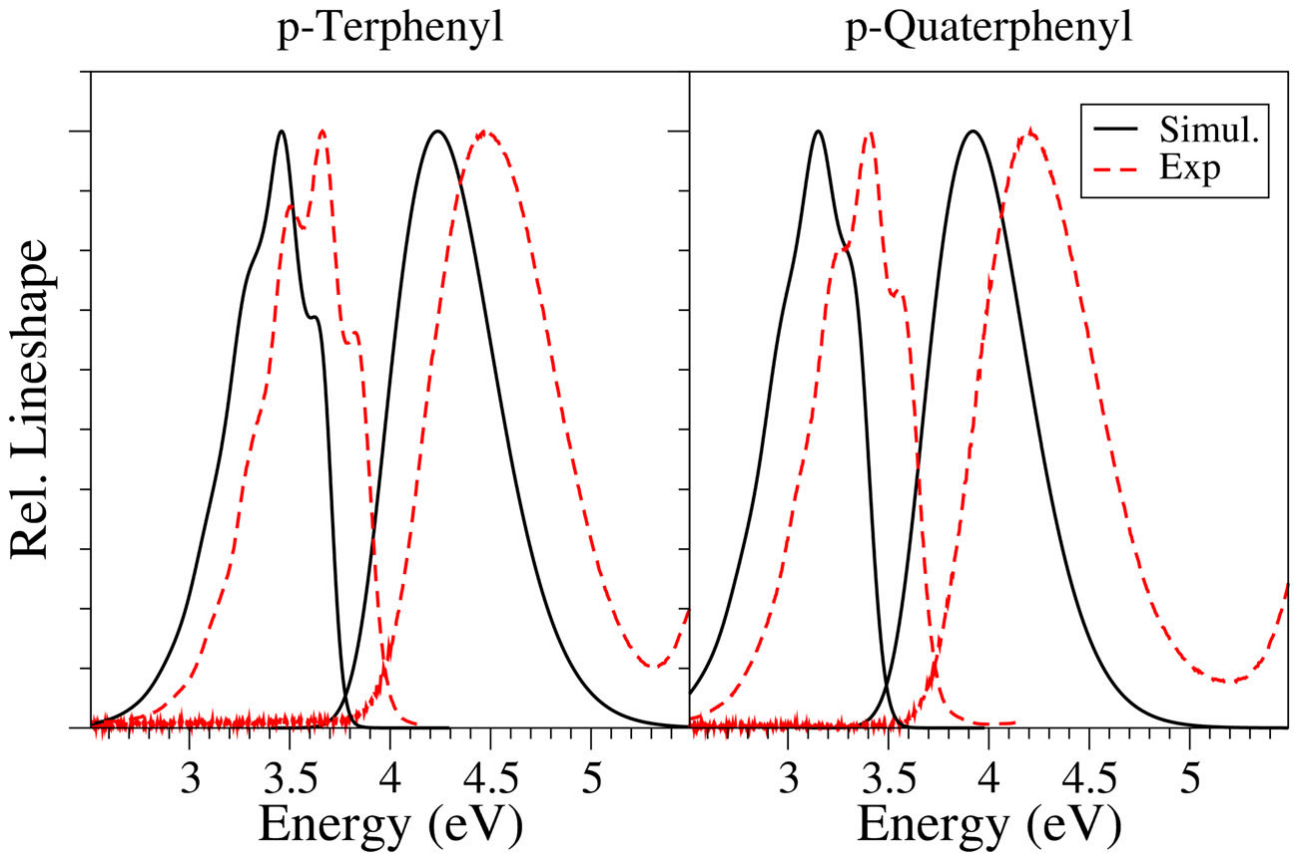
Absorption (top) and emission (bottom) lineshape spectra simulated with the VH model using PBE0. The torsions is removed from the calculation and the effect is recovered by convolution with a Gaussian having the same HWHM as the distributions showing in Figure 8.

In summary, the total spectra are then computed by first building reduced vibrational spaces where the torsions are projected out with the iterative protocols implemented in the code.^56^ In such a space the normal modes with imaginary frequencies present in the full space are removed (2 for p‐terphenyl and 3 for p‐quaterphenyl). Afterward, the contribution of the torsions is included with the convolution with a Gaussian with the computed HWHM. The results are shown in Figure 9, and confirm the good performance of the proposed simple approach. Namely, the spectral shapes generally well reproduced for both emission and absorption. For emission, some small changes when going from p‐terphenyl to p‐quaterphenyl are properly captured, as the relative intensity of the first (almost a shoulder) and second (most intense) peaks. For absorption, the overall width is slightly underestimated. This observation could be partly explained by the expected narrower distributions arising from classical sampling^44^ or by the possible inaccuracies in the excited state PES around the FC point, which reveals critical to determine the spectral width. For instance, Figure S7 shows that using the profile computed for biphenyl to generate the distributions for all the dihedrals of p‐terphenyl and p‐quaterphenyl, the total width of the final spectra changes significantly.

In this section, we illustrated different strategies embedded in *FCclasses3* to deal with a system containing flexible DoFs that compromise the use of the harmonic approach, showing that the use of internal coordinates and projection of coordinates out of the harmonic space within vertical models reveal especially useful in these situations. It is however important to add that the same tools can be applied as steps of more elaborate protocols interfacing *FCclasses3* with other codes. A natural choice, as discussed above is to compute the contributions of torsions at quantum level with a suitable code. It is also noteworthy in this context to mention that, in recent years, we have proposed a method, the adiabatic molecular dynamics generalized vertical Hessian (Ad‐MD|gVH) to simulate the spectroscopy of flexible dyes in explicit environments. According to this method, the spectrum is retrieved as the conformational average (over the solvent and solute flexible DoFs) of vibronic spectra computed by *FCclasses3* for a number of representative snapshots extracted from an MD simulation. More precisely vibronic spectra are computed for reduced‐dimensionality models of the solute obtained removing flexible coordinates and adopting the gVH method.^56^ Convergence typically requires the computation of spectra for a hundred of spectra and this is possible thanks to the effectiveness of the TD approaches implemented in *FCclasses3*.

## 5 CONCLUSIONS

In this contribution, we have presented the new release 3.0 of the freely distributed code *FCclasses*, which has been completely re‐structured, and made more modular, largely extending both the range of systems that can be treated and the number of supported properties. The former goal is achieved by implementing the full family of vertical and adiabatic model harmonic potentials and, mainly, by adopting curvilinear internal coordinates and iterative projectors which leads the code to the limits of applicability of the harmonic approximation. On the one hand, curvilinear coordinates offer a better description of large amplitude motions, which help retain the harmonic approximation. On the other, they provide an excellent framework to build reduced dimensionality spaces, where only stiff (harmonic) modes are kept. In this way, even a very flexible system can be approached, removing the flexible anharmonic modes from the coordinate space adopted in the vibronic calculation. This strategy represents the first step towards designing effective protocols to cope with large, flexible systems.^55^, ^65^, ^94^ Finally, the employment of curvilinear coordinate allows to define vibronic models with expansions at nonstationary points. Besides representing a potentially useful choice in specific cases, this feature opens the route to advanced mixed quantum classical approaches for flexible systems in complex environments, coupling Molecular Dynamics explorations of the configurational space and vibronic computations. Notably, these novel features of *FCclasses3* can be integrated with quantum dynamical propagations over multiple coupled electronic states for treating nonadiabatic systems.^95^

The number of spectroscopic techniques supported by the code has been notably augmented, including also nonlinear and chiroptical ones. To the best of our knowledge, this is the largest set of vibronic spectroscopic simulations available in a single code. All of them are implemented in both TI and TD formulations. The latter allows an efficient calculation of the fully converged spectra, while for former provides information about the nature of the transition responsible for the observed spectral features. The combination of TI and TD calculations reveals as an excellent approach to predict and interpret experimental spectra. In this sense, for properties such as RR, the new availability of the efficient TD implementation allows the detailed exploration of spectral parameters, including the incident frequency and damping factor, at affordable computational cost.

The usability of the program managing such a large amount of properties and options has been facilitated by completely redesigning the input structure and setting sensible defaults for many of the options. In this direction, we provide a new set of tools to generate input data files from output files of different QM codes, or post‐process the results, for instance tuning the broadening of the already computed spectra and helping the visualization and analysis of the transitions from TI calculations or the inspection of RR spectra at varying incident frequencies.

In order to help users to adopt the code for their own purposes, in this new release of the code we put special care in defining transparent standards for feeding information into the code, so to give the possibility to generate them from any source and exploiting different sets of coordinates (normal, Cartesian, internal). At the same time we remarkably increased the number of output data one can get, including for instance, energy and transition amplitude gradients (in normal coordinates), or Duschinsky matrix, for possible uses external to the code. For all the above reasons, we believe that *FCclasses3* represents a useful tool that can be used also by nonexperts in the field. At the same time, it provides some advanced features, such as the generation of reduced‐spaced models, that can be of interest in more focused researches.

The work to further extend the scopes and potentiality of the code will keep on. Possible development routes include, as far as systems are considered, more specialized treatments of multi‐component systems, allowing the definition and projection of intermolecular coordinates. Concerning the available properties we point to implement new nonlinear spectroscopies like for instance resonance hyper‐Raman, or the adoption of cumulant expansions and generalized Brownian oscillator models for simulating bi‐dimensional electronic spectra.^96^ The extended vibrational analysis tools embedded in *FCclasses3* will also be exploited for working out novel advanced tools to investigate and interpret pure vibrational spectroscopies like infrared for complex multichromophoric systems. Moreover, we will also continue our efforts to improve the structure and modularity of the code. Indeed, in the long term, we plan to move towards a plugin‐based paradigm, where the different parts of the code, like the ones dedicated to vibrational analysis, or projectors in curvilinear coordinates, and tools could be called independently from other programs. Finally, regarding the tools, plans involve moving towards popular libraries, such as cclib,^97^ and data formats, such as qcschema,^98^ to extend the interfaces to more QM codes reducing maintenance efforts.

## Supporting information


**Appendix S1:** Supporting Information

**Appendix S2:** Supporting Information

**Video S1:** Supporting Information

## Data Availability

The data that support the findings of this study are available from the corresponding author upon reasonable request.
