# Supplementary material for: FCclasses3: Vibrationally‐resolved spectra simulated at the edge of the harmonic approximation
Source: J Comput Chem. 2022 Nov 15;44(4):626–43. doi: 10.1002/jcc.27027 (PMC10100349; doi:10.1002/jcc.27027)
Supplement: Supplementary file 1 — Appendix S1: Supporting Information [file JCC-44-626-s002.pdf]

FCclasses3: vibrationally resolved electronic spectra at the edge of harmonic approximation (Supporting Information)

Javier Cerezo[a,b] and Fabrizio Santoro[b]

[a] *Departamento de Química* and *Institute for Advanced Research in Chemical Sciences (IAdChem)*,
Universidad Autónoma de Madrid,
Campus de Cantoblanco, 28049 Madrid, Spain

[b] *Istituto di Chimica dei Composti OrganoMetallici*,
Consiglio Nazionale delle Richerche (ICCOM-CNR),
Area della Ricerca, via G. Moruzzi 1, I-56124 Pisa, Italy

November 8, 2022

S1 Iterative propagation of internal coordinates

The iterative protocols introduced in ref. [1] to remove a set of internal coordinates from the harmonic space can be schematiced with the following figure.

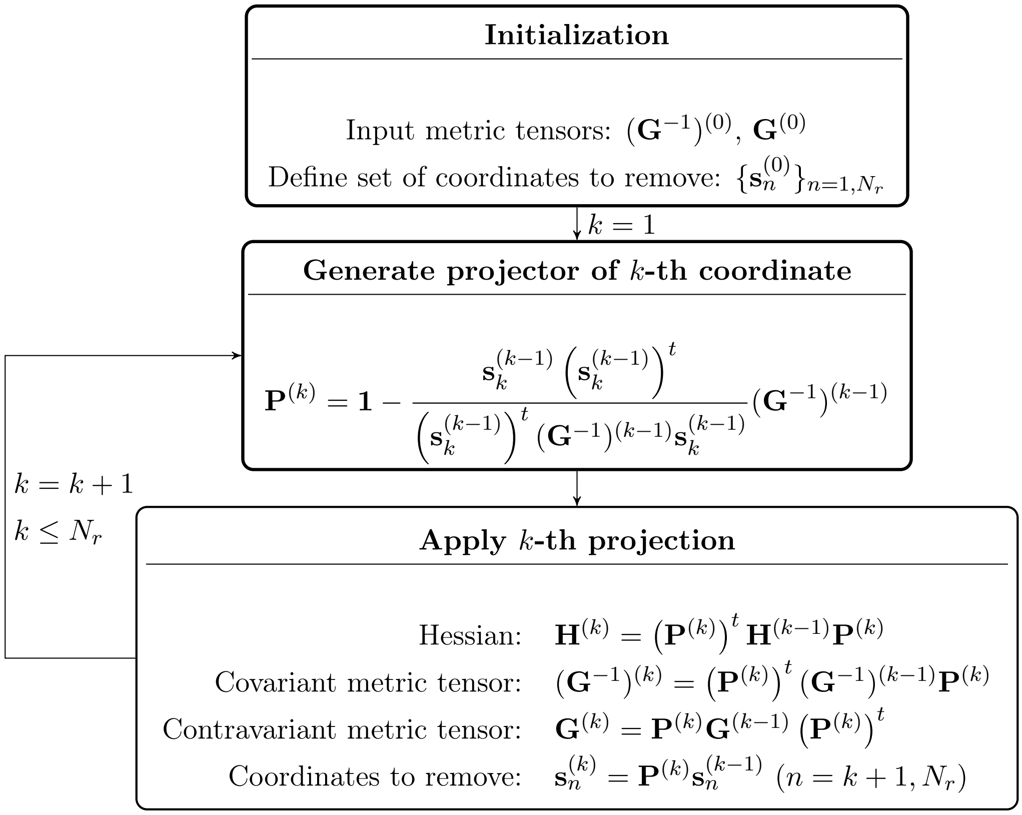

Figure S1: Diagram with the steps to iteratively project out a set of internal coordinates from the Hessian.

S2 Supported properties

As described in Section 2.2 of the manuscript, the code implements a wide range of spectroscopies and non-radiative rates. In that section, the relevant equations for OPA, EMI, ECD and CPL are shown. The expressions implemeted in the code for the rest of spectroscopies are shown below.

S2.1 Magnetic circular dichroism

Vibrationally resolved magnetic circular dichroism due to the Faraday B term can be computed exploiting the same expressions described in the previous section for one-photon spectroscopies[2]. In a TI formalism, the anisotropy of the molar absorptivity for a sample subject to an external magnetic field B_{ext} is

$$\frac{\Delta\epsilon(\omega)}{B_{ext}} = C\omega L(\omega) \tag{S1}$$

where the lineshape in this case is given by,

$$L(\omega) = \sum_{\mathbf{v}_i^g} \sum_{\mathbf{v}_f^f} \rho_{\mathbf{v}_i}(T) B(i\mathbf{v}_i, f\mathbf{v}_f) \delta(\omega_f - \omega_i + (\Delta E/\hbar - \omega)) \tag{S2}$$

The Faraday B term $B(i\mathbf{v}_i, f\mathbf{v}_i f)$ is written as a sum over the vibronic states belonging to excited states different from the initial one. Assuming that the electronic transition frequencies are much larger than vibrational frequencies, an approximated expression, suitable to be adopted in vibronic computations, is obtained[2]:

$$B(i\mathbf{v}_i, f\mathbf{v}_f) = \epsilon_{\alpha\beta\gamma} Im[\langle \mathbf{v}_i | \mu_\alpha^{if} | \mathbf{v}_f \rangle \langle \mathbf{v}_f | T_{\beta\gamma}^{fg} | \mathbf{v}_i \rangle] \tag{S3}$$

where $\epsilon_{\alpha\beta\gamma}$ is the Levi-Civita symbol, and a summation is implicitly involved (Einstein's notation). The tensor T has elements,

$$T_{\beta\gamma}^{fg} = \sum_{k \neq g} \frac{\langle i|\mu_\beta|k\rangle\langle k|m_\gamma|f\rangle}{\omega_k - \omega_g} + \sum_{k \neq f} \frac{\langle i|m_\gamma|k\rangle\langle k|\mu_\beta|f\rangle}{\omega_k - \omega_f} \tag{S4}$$

HT expressions can be obtained by expanding linearly both the Cartesian components of electric transition dipole μ_α^{if} and of the tensor $T_{\beta\gamma}^{fg}$. The formal similarity between

MCD and ECD expressions allows to implement MCD computations in $\mathcal{FC}$*classes3* exactly as for ECD, by simply substituting the values of the magnetic transition dipoles by combinations of the elements of the tensor T^{fg} (and the corresponding derivatives for HT calculations). Further details are given in ref. [2].

S2.2 Two-photon absorption and circular dichroism

The TPA/TPCD intensity and the lineshapes are connected by the following relation,

$$S(\omega) = C\omega^2 L(\omega) \tag{S5}$$

where the expression of the constant C to obtain the spectra in Göppert-Mayer (GM) unit can be found in the literature[3]. The lineshape in the TI formulation leads to the following sum-over-states expression (see ref. [3] and references therein):

$$L(\omega) = \sum_{\mathbf{v}_i}\sum_{\mathbf{v}_f} \rho_{\mathbf{v}_i}(T) D^{i\mathbf{v}_i,f\mathbf{v}_f} \delta(\omega_f - \omega_i + (\Delta E/\hbar - 2\omega)) \tag{S6}$$

where the key quantity is $D^{i\mathbf{v}_i,f\mathbf{v}_f}$ and we make explicit the dependence on both the initial i and final f electronic states.

Both in TPA and TPCD, D depends on the frequency which is approximated to its value at the vertical transition ω_0. In particular in TPA, $D^{i\mathbf{v}_i,f\mathbf{v}_f} = \delta_{TPA}^{i\mathbf{v}_i,f\mathbf{v}_f}(\omega_0)$ and,

$$\delta_{TPA}^{i\mathbf{v}_i,f\mathbf{v}_f}(\omega_0) = \sum_{\alpha,\beta}(F \times S_{\alpha\alpha}^{i\mathbf{v}_i,f\mathbf{v}_f} S_{\beta\beta}^{*,i\mathbf{v}_i,f\mathbf{v}_f} + G \times S_{\alpha\beta}^{i\mathbf{v}_i,f\mathbf{v}_f} S_{\alpha\beta}^{*,i\mathbf{v}_i,f\mathbf{v}_f} + H \times S_{\alpha\beta}^{i\mathbf{v}_i,f\mathbf{v}_f} S_{\beta\alpha}^{*,i\mathbf{v}_i,f\mathbf{v}_f}) \tag{S7}$$

where summation runs over the Cartesian components. In the limit in which the electronic transition frequency is much larger than the vibrational ones,[4] the two-photon transition matrix element $S_{\alpha\beta}^{i\mathbf{v}_i,f\mathbf{v}_f}$ is approximated as $\langle \mathbf{v}_i | S_{\alpha\beta}^{i,f}(\omega_0) | \mathbf{v}_f \rangle$, where

$$S_{\alpha\beta}^{i,f} = \frac{1}{\hbar}\sum_k \frac{\langle i|\mu_\alpha|k\rangle\langle k|\mu_\beta|f\rangle + \langle i|\mu_\beta|k\rangle\langle k|\mu_\alpha|f\rangle}{\omega_k - \omega_0} \tag{S8}$$

In a HT calculation $S_{\alpha\beta}^{i,f}$ is expanded linearly in the normal coordinates of the initial state. The constants F, G and H are 2,2,2 for linearly polarized beams and -2,3,3 for

circularly polarized beams. Further details can be found in ref. [3].

For TPCD in Eq S6 $D^{i\mathbf{v}_i,f\mathbf{v}_f}$ is replaced by the two-photon rotatory strength $R_{TPCD}^{i\mathbf{v}_i,f\mathbf{v}_f}(\omega)$,

$$R_{TPCD}^{i\mathbf{v}_i,f\mathbf{v}_f}(\omega) = -b_1 B_1(\omega) - b_2 B_2(\omega) - b_3 B_3(\omega) \tag{S9}$$

where the constants get the values $b_1 = 6$ and $b_2 = -b_3 = 2$ and we define,

$$B_1(\omega) = \frac{1}{\omega^3} \sum_{\alpha\beta} M_{\alpha\beta}^{p,i\mathbf{v}_i,f\mathbf{v}_f} P^{p,i\mathbf{v}_i,f\mathbf{v}_f,*} \tag{S10}$$

$$B_2(\omega) = \frac{1}{2\omega^3} \sum_{\alpha\beta} T_{\alpha\beta}^{+,i\mathbf{v}_i,f\mathbf{v}_f} P_{\alpha\beta}^{p,i\mathbf{v}_i,f\mathbf{v}_f,*} \tag{S11}$$

$$B_3(\omega) = \frac{1}{\omega^3} \sum_{\alpha\beta} M_{\alpha\alpha}^{p,i\mathbf{v}_i,f\mathbf{v}_f} P_{\beta\beta}^{p,i\mathbf{v}_i,f\mathbf{v}_f,*} \tag{S12}$$

The three tensors M, T^+ and P are described in ref. [3]. Approximations analogous to those employed for TPA lead to convenient expressions for vibronic calculations valid when electronic transition frequencies are much larger than vibrational ones. For instance, for the matrix elements of tensor P, we have $P_{\alpha\beta}^{p,i\mathbf{v}_i,f\mathbf{v}_f,*} \approx \langle \mathbf{v}_i | P_{\alpha\beta}^{i,f}(\omega_0) | \mathbf{v}_f \rangle$ and

$$P_{\alpha\beta}^{i,f}(\omega_0) = \sum_k \frac{\langle i|\mu_\alpha^p|k\rangle\langle k|\mu_\beta^p|f\rangle + \langle i|\mu_\beta^p|k\rangle\langle k|\mu_\alpha^p|f\rangle}{\omega_k - \omega_0} \tag{S13}$$

where μ_α^p is the α Cartesian component of the dipole velocity operator. Also for TPCD, an HT approximation is possible by expanding linearly $P_{\alpha\beta}^{i,f}(\omega_0)$ as a function of the normal coordinates.

An analytical correlation function for this property within a TD formulation can also be derived[5, 6]. In section S3.1, we present the derivation carried out for this work in our group, and implemented in the *$\mathcal{FC}$classes3*. The analytical correlation functions for TPA and TPCD takes a form very similar to those already derived for OPA, and also in these cases they have been implemented including the FC and HT terms.

S2.3 Vibrational Resonance Raman

In vibrational RR spectroscopy, we consider vibrational transitions from the ground (g) vibrational state of the ground electronic state $|\mathbf{0}^g\rangle$, to another vibrational state of the

same electronic state $|\mathbf{v}_f^g\rangle$. The transition polarizability tensor due to monochromatic incident radiation with angular frequency ω_I is

$$\alpha_{\rho\sigma}^{f0} = \frac{1}{\hbar} \sum_{k,\mathbf{v}_m^k} \frac{\langle \mathbf{v}_f^g | \mu_\rho^{gk} | \mathbf{v}_m^k \rangle \langle \mathbf{v}_m^k | \mu_\sigma^{kg} | \mathbf{0}^g \rangle}{\omega_{km,g0} - \omega_I - i\gamma_k} \tag{S14}$$

where the sum is extended in principle to all excited electronic states k and their corresponding vibrational states $|\mathbf{v}_m^k\rangle$, but in practice, it is computed for a particular resonance state. This expression can be formulated in the time-domain exploiting resorting to the relation $1/x = i/\hbar \int_0^\infty dt e^{-ixt/\hbar}$, leading to[7],

$$\alpha_{\rho\sigma}^{f0} = \frac{i}{\hbar^2} \int_0^\infty dt e^{+it\omega_I} e^{-it\Delta E/\hbar} e^{-\gamma_k t} \langle \mathbf{v}_f^g | \mu_\rho^{gk} e^{-i\hat{H}_m t/\hbar} \mu_\sigma^{kg} e^{+i\hat{H}_g t/\hbar} | \mathbf{0}^g \rangle \tag{S15}$$

where $\hat{H}_m$ and $\hat{H}_g$ are the vibrational Hamiltonians for the intermediate and ground states, respectively. The matrix element in bra-ket notation within the time integral can be identified with a correlation function, whose Fourier transform provides the elements of the polarizability tensor. The Cartesian components of the electric transition dipole μ_ρ^{gk} can be expressed as a linear function of the normal coordinates. In that case the polarizability tensor is split in 4 terms "FC-FC", "FC-HT", "HT-FC" and "HT-HT" respectively, which can be evaluated with TI [8] and TD [9, 7, 10] formulations. In the code, we have implemented our own derivation of the TD expression up to overtones and combination bands, which is shown in section S3.2. $\mathcal{FC}$*classes3* computes the RR signal as a two-dimensional spectrum as a function of the incident ω_I and scattered ω_S frequencies. The intensity is computed as $\sigma'(\omega_I, \omega_S)$, the differential cross-section with respect to the scattering solid angle, for a scattered light with any polarization with respect to the scattering plane, collected at 90 degrees with respect to the direction of propagation of the incident light (with perpendicular polarization).[8]

$$\sigma'(\omega_I, \omega_S) = C\omega_S^4 \frac{45a^2 + 7g^2 + 5d^2}{45} \tag{S16}$$

where the rotational invariants a, g and d are respectively the so-called mean polarizability, symmetric anisotropy and antisymmetric anisotropy and are obtained by the polarizability tensors. The expressions for a, g and d and for the constant C can be found in ref. [8]. If N quasi-resonant electronic excited states exist, they all contribute similarly

to the polarizability tensors and interferential effects can emerge in the intensities. They can be described with $\mathcal{FC}$*classes3* adopting a multiple-steps calculation [11]

S2.4 Internal conversion and other non-radiative rates

The non-radiative rate constant is given, following the Fermi golden rule, by:

$$k_{nr} = \frac{2\pi}{\hbar} \sum_{\mathbf{v}_i} \sum_{\mathbf{v}_f} \rho_{\mathbf{v}_i}(T) |\langle \mathbf{v}_i | H'_{if} | \mathbf{v}_f \rangle|^2 \delta(\hbar\omega_f - \hbar\omega_i + \Delta E) \tag{S17}$$

where H'_{if} represents the (small) coupling term that gives rise to the transition between isoenergetic vibrational levels from the initial and final electronic states and, in general, may depend on the nuclear coordinates. The Dirac delta ensures energy conservation considering that ΔE is the adiabatic energy difference between the minima of the initial and final PES, and $\hbar\omega_i$ and $\hbar\omega_f$ are, respectively, the energy of the vibrational states in the initial and final electric states. In practice, the Dirac delta is replaced by a broadening function, $g(\omega)$, which can be either a Gaussian or a Lorentzian (or a combination as in the case Voigt profile). $\rho_{\mathbf{v}_i}(T)$ is the thermal population of the vibrational states in the initial electronic state.

For scalar couplings (intersystem crossings, charge-transfer or exciton transitions), the above expression is similar to that of one-photon lineshapes (Eq. 8 in the main text), setting $\omega = 0$. Therefore, the evaluation of the rate can be carried out with the same TD and TI techniques already implemented for one-photon spectroscopies. Applications of this approach have been published for Charge Transfer rates[12] and intersystem crossings[13].

In the case of internal conversion triggered by non-adiabatic derivative couplings between the initial and final adiabatic states, H'_{if} takes the form,

$$H'_{if} = -\hbar^2 \sum_{k=1}^{N_{vib}} \Lambda_k^{if} \frac{\partial}{\partial Q_k}. \tag{S18}$$

where Λ_k^{if} are the elements of the non-adiabatic coupling vector among electronic states, which can be evaluated with electronic structure methods. Although they may generally depend on nuclear coordinates, we adopt the FC approximation and take them as constant. The TI formulation for the IC rate can then be expressed as,

$$k_{IC} = 2\pi\hbar^3 \sum_{\mathbf{v}_i} \sum_{\mathbf{v}_f} \rho_{\mathbf{v}_i}(T) |\langle \mathbf{v}_i | \mathbf{\Lambda}^{if} \nabla_{\mathbf{Q}} | \mathbf{v}_f \rangle|^2 \delta(\hbar\omega_f - \hbar\omega_i + \Delta E) \quad \text{(S19)}$$

Despite the FC approximation being adopted, the derivative nature of the operator prevents the direct reduction the problem to a simple computation of FC factors, as done above. In the harmonic approximation, the derivative operator can be evaluated adopting second quantization, introducing the momentum operator $\hat{P}_k = -i\hbar\frac{\partial}{\partial Q_k}$. This leads to a problem very similar to that of HT terms for OPA/EMI, which can be solved following similar protocols to evaluate the sum-over-states efficiently (e.g., using the FCclasses algorithm).

In a TD formulations, the IC rate is expressed similarly to the OPA/EMI lineshape (Eq. 9 in the main text), in terms of a correlation function,

$$k_{IC} = \hbar^2 \int dt \; e^{-it(\Delta E/\hbar)} \chi^{IC}(t, T) g'(t) \quad \text{(S20)}$$

where $g'(t)$ is a damping function and χ^{IC} is a correlation function that reads,

$$\chi(t, T) = Z_{vib}^{-1} \mathrm{Tr} \left[\mathbf{\Lambda}^{if} \nabla_{\mathbf{Q}} e^{-i\hat{H}_f \tau_f} \mathbf{\Lambda}^{if} \nabla_{\mathbf{Q}} e^{-i\hat{H}_i \tau_i} \right] \quad \text{(S21)}$$

where $\tau_f = t/\hbar$ and $\tau_i = -i\beta - t/\hbar$, and $\hat{H}_i$ and $\hat{H}_f$ are the vibrational (harmonic) Hamiltonians for the initial and final states. The above correlation function can be evaluated analytically as shown in refs. [14, 15, 16]. The calculation eventually provides a spectrum-like object, with the values of k_{IC} as function of the energy difference between the initial and final PES minima. The searched value k_{IC} must be taken at the specific ΔE value but evaluation of the full profile can be interesting to check the quality of the results.[17] Since in many cases ΔE is large (especially when the final state of the transition is the ground-electronic state), the actual value of the rate is actually taken in the very tail of the k_{IC}. This is the part where TI calculation usually suffers from convergence problems, limiting its applicability for already moderate ΔE values (> 1.5-2 eV), making TD formulation the method of choice for these type of calculations[17]. In any case, for large ΔE caution is still needed even in the TD implementation when using a Lorentzian broadening, since, due to its slow-decaying tail, it can dominate the estimate of the rate, making it unreliable. It should be further warned that for large

ΔE the reliability of harmonic approximation can be questioned. A detailed discussion of these issues can be found in ref. [17].

S3 Other implementation details

S3.1 Correlation function for two-photon absorption

To get a working expression we need first to plug the expression for δ_{TPA} (19) into that of the two-photon lineshape (18), approximating $S_{\alpha\beta}^{i\mathbf{v}_i,f\mathbf{v}_f}$ by $\langle \mathbf{v}_i|S_{\alpha\beta}^{i,f}(\omega_0)|\mathbf{v}_f\rangle$. We then transform the Dirac delta (or the convenient broadening function) with the Fourier Transform, i.e., $\delta(\omega_f - \omega_i + \Delta E/\hbar - 2\omega) = 1/2\pi \int dt e^{-it(\omega_f-\omega_i+\Delta E/\hbar-2\omega)}$ and consider that $\rho_i = Z_{vib}^{-1} e^{-\beta\hbar\omega_i}$ to yield (note we drop the explicit mention to ω_0 dependency),

$$L(\omega) = \frac{1}{Z_{vib}2\pi}\int_{-\infty}^{\infty} dt \sum_{\mathbf{v}_i}\sum_{\mathbf{v}_f} e^{-\beta\hbar\omega_i} e^{-it\omega_f} e^{+it\omega_i} e^{-it(\Delta E/\hbar-2\omega)}$$
$$\left\{\sum_{\alpha,\beta} F\langle \mathbf{v}_i|S_{\alpha\alpha}^{i,f}|\mathbf{v}_f\rangle\langle \mathbf{v}_f|S_{\beta\beta}^{i,f}|\mathbf{v}_i\rangle + G\langle \mathbf{v}_i|S_{\alpha\beta}^{i,f}|\mathbf{v}_f\rangle\langle \mathbf{v}_f|S_{\alpha\beta}^{i,f}|\mathbf{v}_i\rangle + H\langle \mathbf{v}_i|S_{\alpha\beta}^{i,f}|\mathbf{v}_f\rangle\langle \mathbf{v}_f|S_{\beta\alpha}^{i,f}|\mathbf{v}_i\rangle\right\} \tag{S22}$$

We now introduce $\tau_f = t/\hbar$ and $\tau_i = -i\beta - t/\hbar$ and reorganize the exponentials,

$$\begin{aligned} L(\omega) = & \frac{1}{Z_{vib}2\pi}\int_{-\infty}^{\infty} dt e^{-it(\Delta E/\hbar-2\omega)}\times \\ & \left\{\sum_{\alpha,\beta} F\sum_{\mathbf{v}_i}\sum_{\mathbf{v}_f}\langle \mathbf{v}_i|S_{\alpha\alpha}^{i,f}e^{-i\hbar\omega_f\tau_f}|\mathbf{v}_f\rangle\langle \mathbf{v}_f|S_{\beta\beta}^{i,f}e^{-i\hbar\omega_i\tau_i}|\mathbf{v}_i\rangle + \right. \\ & G\sum_{\mathbf{v}_i}\sum_{\mathbf{v}_f}\langle \mathbf{v}_i|S_{\alpha\beta}^{i,f}e^{-i\hbar\omega_f\tau_f}|\mathbf{v}_f\rangle\langle \mathbf{v}_f|S_{\alpha\beta}^{i,f}e^{-i\hbar\omega_i\tau_i}|\mathbf{v}_i\rangle + \\ & \left. H\sum_{\mathbf{v}_i}\sum_{\mathbf{v}_f}\langle \mathbf{v}_i|S_{\alpha\beta}^{i,f}e^{-i\hbar\omega_f\tau_f}|\mathbf{v}_f\rangle\langle \mathbf{v}_f|S_{\beta\alpha}^{i,f}e^{-i\hbar\omega_i\tau_i}|\mathbf{v}_i\rangle\right\} \end{aligned} \tag{S23}$$

The term in curly braces correspond to the correlation function whose Fourier Transform would lead to the lineshape, $\chi_{TPA}(t,T)$. Further recognizing that $\hbar\omega_i$ and $\hbar\omega_f$ are the eigenvalues of the nuclear Hamiltonian operators for the initial and final states, and exploiting the closure relation for the final state, $\sum_{\mathbf{v}_f}|\mathbf{v}_f\rangle\langle\mathbf{v}_f| = 1$, the correlation function can be expressed as,

$$\chi^{TPA}(t,T) = \sum_{\alpha,\beta} \begin{array}{l} F\sum_{\mathbf{v}_i}\langle \mathbf{v}_i|S^{i,f}_{\alpha\alpha}e^{-i\hbar\omega_f\tau_f}S^{i,f}_{\beta\beta}e^{-i\hbar\omega_i\tau_i}|\mathbf{v}_i\rangle + \\ G\sum_{\mathbf{v}_i}\langle \mathbf{v}_i|S^{i,f}_{\alpha\beta}e^{-i\hbar\omega_f\tau_f}S^{i,f}_{\alpha\beta}e^{-i\hbar\omega_i\tau_i}|\mathbf{v}_i\rangle + \\ H\sum_{\mathbf{v}_i}\langle \mathbf{v}_i|S^{i,f}_{\alpha\beta}e^{-i\hbar\omega_f\tau_f}S^{i,f}_{\beta\alpha}e^{-i\hbar\omega_i\tau_i}|\mathbf{v}_i\rangle \end{array} \quad \text{(S24)}$$

We now adopt FC and HT terms to describe the dependency of electronic tensor elements in terms of nuclear coordinates,

$$S^{i,f}_{\alpha\beta}(\mathbf{Q}) = S^{i,f}_{\alpha\beta}(\mathbf{Q}_0) + \sum_{k=1}^{N_{vib}} \frac{\partial S^{i,f}_{\alpha\beta}(\mathbf{Q})}{\partial Q_k} Q_k \equiv S^{i,f,(0)}_{\alpha\beta} + \sum_{k=1}^{N_{vib}} S^{i,f,(1)}_{\alpha\beta,k} Q_k \quad \text{(S25)}$$

where we introduce a simplified nomenclature for the value of the element at the reference geometry ($S^{i,f,(0)}_{\alpha\beta}$) and its first derivative with respect to k-th coordinate ($S^{i,f,(1)}_{\alpha\beta,k}$). N_vib is the number of vibrational coordinates.

Substituting that expression in Eq. (S24) and rearranging we arrive to,

$$\chi^{TPA}(t,T) = \chi_{FC}(t,T) + \chi_{FC/HT}(t,T) + \chi_{HT/HT}(t,T) \quad \text{(S26)}$$

where each term is given by,

$$\chi_{FC}(t,T) = \left[\sum_{\alpha\beta} F\cdot S^{i,f,(0)}_{\alpha\alpha}S^{i,f,(0)}_{\beta\beta} + G\cdot S^{i,f,(0)}_{\alpha\beta}S^{i,f,(0)}_{\alpha\beta} + H\cdot S^{i,f,(0)}_{\alpha\beta}S^{i,f,(0)}_{\beta\alpha}\right] \int_{\mathbf{Q}} d\mathbf{Q}\langle\mathbf{Q}|e^{-itH_f/\hbar}e^{-(\beta-it)H_i/\hbar}|\mathbf{Q}\rangle \tag{S27a}$$

$$\begin{aligned}\chi_{FC/HT}(t,T) = \sum_k \left[\sum_{\alpha\beta} F\cdot S^{i,f,(1)}_{\alpha\alpha,k}S^{i,f,(0)}_{\beta\beta} + G\cdot S^{i,f,(1)}_{\alpha\beta,k}S^{i,f,(0)}_{\alpha\beta} + H\cdot S^{i,f,(1)}_{\alpha\beta,k}S^{i,f,(0)}_{\beta\alpha}\right] \\ \int_{\mathbf{Q}} d\mathbf{Q}\langle\mathbf{Q}|Q_k e^{-itH_f/\hbar}e^{-(\beta-it)H_i/\hbar}|\mathbf{Q}\rangle + \\ \sum_k \left[\sum_{\alpha\beta} F\cdot S^{i,f,(0)}_{\alpha\alpha}S^{i,f,(1)}_{\beta\beta,k} + G\cdot S^{i,f,(0)}_{\alpha\beta}S^{i,f,(1)}_{\alpha\beta,k} + H\cdot S^{i,f,(0)}_{\alpha\beta}S^{i,f,(1)}_{\beta\alpha,k}\right] \\ \int_{\mathbf{Q}} d\mathbf{Q}\langle\mathbf{Q}|e^{-itH_f/\hbar}Q_k e^{-(\beta-it)H_i/\hbar}|\mathbf{Q}\rangle\end{aligned} \tag{S27b}$$

$$\chi_{HT/HT}(t,T) = \left[\sum_{\alpha\beta} F\cdot S^{i,f,(1)}_{\alpha\alpha,k}S^{i,f,(1)}_{\beta\beta,l} + G\cdot S^{i,f,(1)}_{\alpha\beta,k}S^{i,f,(1)}_{\alpha\beta,l} + H\cdot S^{i,f,(1)}_{\alpha\beta,k}S^{i,f,(1)}_{\beta\alpha,l}\right] \int_{\mathbf{Q}} d\mathbf{Q}\langle\mathbf{Q}|Q_k e^{-itH_f/\hbar}Q_l e^{-(\beta-it)H_i/\hbar}|\mathbf{Q}\rangle \tag{S27c}$$

where k and l index in the sums run over the N_{vib} vibrational coordinates and we have evaluated the trace over the coordinate space, instead of the vibrational wavefunction space, i.e., $\mathrm{Tr}[\hat{A}] = \sum_{\mathbf{v}_i}\langle\mathbf{v}_i|\hat{A}|\mathbf{v}_i\rangle = \int d\mathbf{Q}\langle\mathbf{Q}|\hat{A}|\mathbf{Q}\rangle$ The analytical solution of the integrals can be carried out[18, 14], to provide the following expression for the total correlation function,

$$\chi^{tot}(t,T) = \chi^{FC}(t,T)\left(\mathcal{S}^{(0,0)} + \sum_k\left[\mathcal{S}^{(1,0)}_k + \mathcal{S}^{(0,1)}_k\right](D_{HT})_k + \sum_{k,l}\mathcal{S}^{(1,1)}_{k,l}(A_{HT})_{kl}\right) \tag{S28}$$

where k and l indexes in the sums run over the N_{vib} vibrational coordinates and we have

introduced the elements,

$$\mathcal{S}^{(0,0)} = \sum_{\alpha\beta} F \cdot S^{i,f,(0)}_{\alpha\alpha} S^{i,f,(0)}_{\beta\beta} + G \cdot S^{i,f,(0)}_{\alpha\beta} S^{i,f,(0)}_{\alpha\beta} + H \cdot S^{i,f,(0)}_{\alpha\beta} S^{i,f,(0)}_{\beta\alpha} \tag{S29a}$$

$$\mathcal{S}^{(1,0)}_{k} = \sum_{\alpha\beta} F \cdot S^{i,f,(1)}_{\alpha\alpha,k} S^{i,f,(0)}_{\beta\beta} + G \cdot S^{i,f,(1)}_{\alpha\beta,k} S^{i,f,(0)}_{\alpha\beta} + H \cdot S^{i,f,(1)}_{\alpha\beta,k} S^{i,f,(0)}_{\beta\alpha} \tag{S29b}$$

$$\mathcal{S}^{(0,1)}_{k} = \sum_{\alpha\beta} F \cdot S^{i,f,(0)}_{\alpha\alpha} S^{i,f,(1)}_{\beta\beta,k} + G \cdot S^{i,f,(0)}_{\alpha\beta} S^{i,f,(1)}_{\alpha\beta,k} + H \cdot S^{i,f,(0)}_{\alpha\beta} S^{i,f,(1)}_{\beta\alpha,k} \tag{S29c}$$

$$\mathcal{S}^{(1,1)}_{k,l} = \sum_{\alpha\beta} F \cdot S^{i,f,(1)}_{\alpha\alpha,k} S^{i,f,(1)}_{\beta\beta,l} + G \cdot S^{i,f,(1)}_{\alpha\beta,k} S^{i,f,(1)}_{\alpha\beta,l} + H \cdot S^{i,f,(1)}_{\alpha\beta,k} S^{i,f,(1)}_{\beta\alpha,l} \tag{S29d}$$

The FC correlation function, $\chi^{FC}(t,T)$, is given by,

$$\chi^{FC}(t,T) = \sqrt{\frac{\det(\mathbf{a_f})\det(\mathbf{a_i})}{(i\hbar)^{2N}\det(\mathbf{C})\det(\mathbf{D})}} \times \exp\left[-\mathbf{K}^T\mathbf{d_i}\mathbf{K} + \mathbf{K}^T\mathbf{d_i}\mathbf{J}\mathbf{D^{-1}}\mathbf{J^T}\mathbf{d_i}\mathbf{K}\right] \tag{S30}$$

The $\mathbf{a}_\alpha$, $\mathbf{a}_\alpha$, $\mathbf{c}_\alpha$ and $\mathbf{d}_\alpha$ (where $\alpha = \mathbf{i},\mathbf{f}$) diagonal matrices depend on the time, and they are defined with elements,

$$(a_\alpha(\tau))_{kk} = \frac{\omega_k}{\sin(\hbar\omega_k\tau)} \tag{S31a}$$

$$(c_\alpha(\tau))_{kk} = \frac{\omega_k}{\coth(i\hbar\omega_k\tau/2)} \tag{S31b}$$

$$(d_\alpha(\tau))_{kk} = \frac{\omega_k}{\tanh(i\hbar\omega_k\tau/2)} \tag{S31c}$$

where $\alpha = \mathbf{i},\mathbf{f}$ refers to the either initial (i) or final (f) states, ω_k is the frequency of each harmonic oscillator and the τ is defined as $\tau_i = -t - i\beta$ and $\tau_f = t$ for the initial and final states, respectively. From these ones, the following matrices, also depending on time, are defined,

$$\mathbf{C}(\tau_i,\tau_f) = \mathbf{c_f}(\tau_f) + \mathbf{J^T}\mathbf{c_i}(\tau_i)\mathbf{J} \tag{S32a}$$

$$\mathbf{D}(\tau_i,\tau_f) = \mathbf{d_f}(\tau_f) + \mathbf{J^T}\mathbf{d_i}(\tau_i)\mathbf{J} \tag{S32b}$$

$$\mathbf{D_{HT}} = -\mathbf{D}^{-1}\mathbf{J}^T\mathbf{d_i}\mathbf{K} \tag{S32c}$$

$$\mathbf{A_{HT}} = \mathbf{D_{HT}}\mathbf{D}^T_{\mathbf{HT}} + \frac{1}{2}(\mathbf{D}^{-1} - \mathbf{C}^{-1}) \tag{S32d}$$

An analogous expression is derived for TPCD, by appropriately changing the definition of the $\mathcal{S}$ matrix elements as follows,

$$\mathcal{S}^{(0,0)}_{TPCD} = \frac{-1}{\omega^3}\sum_{\alpha\beta} b_3 \cdot M^{i,f,(0)}_{\alpha\alpha} P^{i,f,(0)}_{\beta\beta} + \frac{b_2}{2} \cdot T^{+,i,f,(0)}_{\alpha\beta} P^{i,f,(0)}_{\alpha\beta} + b_1 \cdot M^{i,f,(0)}_{\alpha\beta} P^{i,f,(0)}_{\beta\alpha} \tag{S33a}$$

$$\mathcal{S}^{(1,0)}_{TPCD;k} = \frac{-1}{\omega^3}\sum_{\alpha\beta} b_3 \cdot M^{i,f,(1)}_{\alpha\alpha,k} P^{i,f,(0)}_{\beta\beta} + \frac{b_2}{2} \cdot T^{+,i,f,(1)}_{\alpha\beta,k} P^{i,f,(0)}_{\alpha\beta} + b_1 \cdot M^{i,f,(1)}_{\alpha\beta,k} P^{i,f,(0)}_{\beta\alpha} \tag{S33b}$$

$$\mathcal{S}^{(0,1)}_{TPCD;k} = \frac{-1}{\omega^3}\sum_{\alpha\beta} b_3 \cdot M^{i,f,(0)}_{\alpha\alpha} P^{i,f,(1)}_{\beta\beta,k} + \frac{b_2}{2} \cdot T^{+,i,f,(0)}_{\alpha\beta} P^{i,f,(1)}_{\alpha\beta,k} + b_1 \cdot M^{i,f,(0)}_{\alpha\beta} P^{i,f,(1)}_{\beta\alpha,k} \tag{S33c}$$

$$\mathcal{S}^{(1,1)}_{TPCD;k,l} = \frac{-1}{\omega^3}\sum_{\alpha\beta} b_3 \cdot M^{i,f,(1)}_{\alpha\alpha,k} P^{i,f,(1)}_{\beta\beta,l} + \frac{b_2}{2} \cdot T^{+,i,f,(1)}_{\alpha\beta,k} P^{i,f,(1)}_{\alpha\beta,l} + b_1 \cdot M^{i,f,(1)}_{\alpha\beta,k} P^{i,f,(1)}_{\beta\alpha,l} \tag{S33d}$$

where the tensors M, T^+ and P and the constants b_1, b_2 and b_3, are those referenced in the manuscript.

S3.2 Correlation function for Resonant Raman spectroscopy

The elements of the polarizability tensor at 0 K can be computed from,

$$\alpha^{f0}_{\rho\sigma} = \frac{i}{\hbar^2}\int_0^\infty dt e^{+it\omega_I} e^{-it\Delta E/\hbar} e^{-\gamma_k t} \chi^{v^g_f}_{\rho\sigma}(t,T) \tag{S34}$$

where the following correlation function for the (ρ,σ) element was introduced,

$$\chi^{|\mathbf{v}^g_f\rangle}_{\rho\sigma}(t,T) = \langle \mathbf{v}^g_f | \mu^{gk}_\rho e^{-i\hat{H}_m t/\hbar} \mu^{kg}_\sigma e^{+i\hat{H}_g t/\hbar} | \mathbf{0}^g \rangle = e^{iE_0 t/\hbar} \langle \mathbf{v}^g_f | \mu^{gk}_\rho e^{-i\hat{H}_m t/\hbar} \mu^{kg}_\sigma | \mathbf{0}^g \rangle \tag{S35}$$

where $E_0 = \frac{1}{2}\hbar\sum_{i=1}^{N_{vib}}\omega_i$

If we now add two complete sets of normal mode coordinates in the initial and final states,

$$\chi_{\rho\sigma}^{|\mathbf{v}_f^g\rangle}(t,T) = e^{iE_0 t/\hbar}\int_{-\infty}^{\infty}\int_{-\infty}^{\infty}\int_{-\infty}^{\infty}\int_{-\infty}^{\infty} d\mathbf{Q}_f d\bar{\mathbf{Q}}_f d\mathbf{Q}_i d\bar{\mathbf{Q}}_i$$
$$\langle \mathbf{v}_f^g|\mathbf{Q}_i\rangle\langle \mathbf{Q}_i|\mu_\rho^{gk}|\mathbf{Q}_f\rangle\langle \mathbf{Q}_f|e^{-i\hat{H}_m t/\hbar}|\bar{\mathbf{Q}}_f\rangle\langle \bar{\mathbf{Q}}_f|\mu_\sigma^{kg}|\bar{\mathbf{Q}}_i\rangle\langle \bar{\mathbf{Q}}_i|\mathbf{0}^g\rangle \tag{S36}$$

The elements μ_ρ^{gk} and μ_σ^{kg} generally depend on nuclear coordinates, and they can be expanded as Taylor series in terms of $\mathbf{Q}_f$

$$\mu_\rho^{gk}(\mathbf{Q}) = \mu_\rho^{gk}(\mathbf{Q}_0) + \sum_{k=1}^{N_{vib}} \frac{\partial \mu_\rho^{gk}(\mathbf{Q})}{\partial Q_f} Q_f \equiv \mu_\rho^{gk,(0)} + \sum_{k=1}^{N_{vib}} \mu_{\rho,k}^{gk,(1)} Q_f \tag{S37}$$

where both the constant (FC) and linear (HT) terms are included, and a simplified notation to represent the first derivatives is introduced. Using the Duschinsky relation between the normal modes in the initial and final states, $\mathbf{Q}_i = \mathbf{J}\mathbf{Q}_f + \mathbf{K}$, we can write:

$$\langle \mathbf{Q}_i|\mu_\rho^{gk}(\mathbf{Q}_f)|\mathbf{Q}_f\rangle = \mu_\rho^{gk}(\mathbf{Q}_f)\delta(\mathbf{Q}_i - \mathbf{J}\mathbf{Q}_f + \mathbf{K}) \tag{S38a}$$
$$\langle \bar{\mathbf{Q}}_f|\mu_\sigma^{kg}(\mathbf{Q}_f)|\bar{\mathbf{Q}}_i\rangle = \mu_\sigma^{kg}(\bar{\mathbf{Q}}_f)\delta(\bar{\mathbf{Q}}_i - \mathbf{J}\bar{\mathbf{Q}}_f + \mathbf{K}) \tag{S38b}$$

The projection of the vibrational eigenstates in the coordinate space corresponds to the vibrational wavefunctions. Focusing on the Rayleigh ($|\mathbf{v}_f^g\rangle = |\mathbf{0}^g\rangle$) and fundamental ($|\mathbf{v}_f^g\rangle = |\mathbf{0}^g + 1_k^g\rangle$, with $k = 1, N_{vib}$), the relevant wavefunctions are,

$$\langle \mathbf{0}^g + 1_k^g|\mathbf{Q}_i\rangle = \sqrt{2\Gamma_{ik}}Q_{ik}\frac{\det[\mathbf{\Gamma}_i]}{\pi^{N/4}}e^{-\frac{1}{2}\mathbf{Q}_i^t\mathbf{\Gamma}_i\mathbf{Q}_i} \tag{S39a}$$
$$\langle \mathbf{0}^g|\mathbf{Q}_i\rangle = \frac{\det[\mathbf{\Gamma}_i]}{\pi^{N/4}}e^{-\frac{1}{2}\mathbf{Q}_i^t\mathbf{\Gamma}_i\mathbf{Q}_i} \tag{S39b}$$
$$\langle \bar{\mathbf{Q}}_i|\mathbf{0}^g\rangle = \frac{\det[\mathbf{\Gamma}_i]}{\pi^{N/4}}e^{-\frac{1}{2}\bar{\mathbf{Q}}_i^t\mathbf{\Gamma}_i\bar{\mathbf{Q}}_i} \tag{S39c}$$

where $\mathbf{\Gamma}_i$ is a diagonal matrix with elements $\Gamma_{i,kk} = \omega_{i,k}/\hbar$.

Finally, we exploit the analytical expression for the off-diagonal matrix elements evaluated within the Path Integral formulation by Feynman,

$$\langle \mathbf{Q}_f|e^{-i\hat{H}_f\tau}|\bar{\mathbf{Q}}_f\rangle = \left(\frac{\det[\mathbf{a}_f(\tau)]}{(2\pi i\hbar)^N}\right)^{1/2} e^{\frac{i}{\hbar}\left[\frac{1}{2}\mathbf{Q}_f^t\mathbf{b}_f\mathbf{Q}_f + \frac{1}{2}\bar{\mathbf{Q}}_f^t\mathbf{b}_f\bar{\mathbf{Q}}_f - \mathbf{Q}_f^t\mathbf{a}_f\bar{\mathbf{Q}}_f\right]} \tag{S40}$$

where **a** and **b** are diagonal matrices, with elements,

$$(a_f(\tau))_{kk} = \frac{\omega_{f,k}}{\sin(\hbar\omega_{f,k}\tau)} \tag{S41a}$$

$$(b_f(\tau))_{kk} = \frac{\omega_{f,k}}{\tan(\hbar\omega_{f,k}\tau)} \tag{S41b}$$

Plugging the above expressions into Eq. S36, considering that $e^{iE_0t/\hbar} = \frac{1}{\det[e^{-i\hbar\boldsymbol{\Gamma}_i}]^{1/2}}$, and after some manipulations (see Refs. [19]), the correlation function can be expressed as,

$$\begin{aligned}\chi_{\rho\sigma} = \left(\frac{\det(\mathbf{a_f})\det(\mathbf{a_i}')}{(i\hbar)^{2N}}\right)^{1/2} e^{\frac{i}{\hbar}\mathbf{K}^t\mathbf{E}\mathbf{K}} \int_{-\infty}^{\infty}\int_{-\infty}^{\infty} d\mathbf{Q}_f d\bar{\mathbf{Q}}_f \\ \mu_\rho^{gk}(\mathbf{Q}_f)\mu_\sigma^{gk}(\bar{\mathbf{Q}}_f)F_{v_f^g}(\mathbf{Q}_f) \\ \exp\left[\frac{i}{\hbar}\left(\frac{1}{2}\mathbf{Q}_f^t\mathbf{B}\mathbf{Q}_f + \frac{1}{2}\bar{\mathbf{Q}}_f^t\mathbf{B}\bar{\mathbf{Q}}_f + \mathbf{K}^t\mathbf{E}\mathbf{J}(\mathbf{Q}_f + \bar{\mathbf{Q}}_f) - \mathbf{Q}_f^t\mathbf{A}\bar{\mathbf{Q}}_f\right)\right]\end{aligned} \tag{S42}$$

where the factor $F_{v_f^g}(\mathbf{Q}_f)$ is 1 for the Rayleigh band and $\sqrt{2\Gamma_{ik}}(\boldsymbol{\alpha}\mathbf{Q}_f + K_k)$ for fundamental bands. We have introduced $\boldsymbol{\alpha}$ vector, with elements $\alpha_l \equiv J_{kl}$, and J_{kl} and K_k represent elements of the Duschinsky matrix ($\mathbf{J}$) and normal mode displacement vector ($\mathbf{K}$), respectively. Moreover, the following matrices are introduced,

$$\mathbf{a_i'} = \frac{2i\hbar\boldsymbol{\Gamma}_i}{e^{-2i\hbar\boldsymbol{\Gamma}_i t}} \tag{S43a}$$

$$\mathbf{A} = \mathbf{a_f} \tag{S43b}$$

$$\mathbf{B} = \mathbf{b_f} - \mathbf{J}^t(i\hbar\boldsymbol{\Gamma}_i)\mathbf{J} \tag{S43c}$$

$$\mathbf{E} = i\hbar\boldsymbol{\Gamma}_i \tag{S43d}$$

The above Gaussian integral can be solved[19] by first applying the change of variable,

$$\mathbf{Z} = \frac{1}{\sqrt{2}}(\mathbf{Q}_f + \bar{\mathbf{Q}}_f)\mathbf{U} = \frac{1}{\sqrt{2}}(\mathbf{Q}_f - \bar{\mathbf{Q}}_f) \tag{S44}$$

which decouples all coordinates in the integrand, followed by an additional change of

variable to get Gaussian integrals in the standard form,

$$\mathbf{Z}_1 = \mathbf{D}^{1/2}\mathbf{Z} + \sqrt{2}\mathbf{D}^{-1/2}\mathbf{J}^t\mathbf{d}_i\mathbf{K} = \mathbf{D}^{1/2}\mathbf{Z} + \sqrt{2}\mathbf{D}^{-1/2}\boldsymbol{\lambda} \tag{S45a}$$

$$\mathbf{U}_1 = \mathbf{C}^{1/2}\mathbf{U} \tag{S45b}$$

where we have introduced the diagonal matrix $\mathbf{d}_i$ with elements $d_{i,kk} = \omega_{g,k}$ and the matrices,

$$\mathbf{C} = -\frac{i}{\hbar}(\mathbf{B} + \mathbf{A}) \tag{S46a}$$

$$\mathbf{D} = -\frac{i}{\hbar}(\mathbf{B} - \mathbf{A}) \tag{S46b}$$

(S46c)

After such transformations [19], the integral is simplified to,

$$\chi_{\rho\sigma} = \chi_0 \frac{1}{(2\pi)^N} \int_{-\infty}^{\infty}\int_{-\infty}^{\infty} d\mathbf{Z}_1 d\mathbf{U}_1 \exp\left[-\frac{1}{2}\mathbf{Z}_1^t\mathbf{Z}_1\right] \exp\left[-\frac{1}{2}\mathbf{U}_1^t\mathbf{U}_1\right] \\ \mu_\rho^{gk}(\mathbf{Z}_1, \mathbf{U}_1)\mu_\sigma^{gk}(\mathbf{Z}_1, \mathbf{U}_1)F_{v_f^g}(\mathbf{Z}_1, \mathbf{U}_1) \tag{S47}$$

where we have indicated that μ_ρ^{gk}, μ_σ^{gk} and $F_{v_f^g}$ are expressed in terms of the new variables, $\mathbf{Z}_1, \mathbf{U}_1$, and we grouped the common integral prefactor in χ_0, which reads,

$$\chi_0 = \sqrt{\frac{\det(\mathbf{a_f})\det(\mathbf{a_i}')}{(i\hbar)^{2N}\det(\mathbf{C})\det(\mathbf{D})}} \times \exp\left[-\mathbf{K}^T\mathbf{d_i}\mathbf{K} + \mathbf{K}^T\mathbf{d_i}\mathbf{J}\mathbf{D^{-1}}\mathbf{J^T}\mathbf{d_i}\mathbf{K}\right] \tag{S48}$$

The expressions for the transition dipoles in terms of the new coordinates, $\mathbf{Z}_1$, $\mathbf{U}_1$, read,

$$\mu_\rho^{gk}(\mathbf{Q}_f) = \mu_\rho^{gk,0} + (\boldsymbol{\mu}_\rho^{(1)})^t\mathbf{Q}_f = \mu_\rho^{gk,0} + \frac{\sqrt{2}}{2}(\boldsymbol{\mu}_\rho^{(1)})^t\mathbf{D}^{-1/2}\mathbf{Z}_1 + \frac{\sqrt{2}}{2}(\boldsymbol{\mu}_\rho^{(1)})^t\mathbf{C}^{-1/2}\mathbf{U}_1 + (\boldsymbol{\mu}_\rho^{(1)})^t\mathbf{D}_{HT} \tag{S49a}$$

$$\mu_\sigma^{kg}(\bar{\mathbf{Q}}_f) = \mu_\sigma^{kg,0} + (\boldsymbol{\mu}_\sigma^{(1)})^t\bar{\mathbf{Q}}_f = \mu_\sigma^{kg,0} + \frac{\sqrt{2}}{2}(\boldsymbol{\mu}_\sigma^{(1)})^t\mathbf{D}^{-1/2}\mathbf{Z}_1 - \frac{\sqrt{2}}{2}(\boldsymbol{\mu}_\sigma^{(1)})^t\mathbf{C}^{-1/2}\mathbf{U}_1 + (\boldsymbol{\mu}_\sigma^{(1)})^t\mathbf{D}_{HT} \tag{S49b}$$

where $\mathbf{D}_{HT} = -\mathbf{D}^{-1}\boldsymbol{\lambda}$, with $\boldsymbol{\lambda} = \mathbf{K}^t\mathbf{d}_i\mathbf{J}$. Multyplying both terms we arrive to the general expression,

$$\mu_\rho^{gk}\mu_\sigma^{kg} = \mathbf{Y}^0 + \mathbf{Y}^Z\mathbf{Z}_1 + \mathbf{Y}^U\mathbf{U}_1 + \mathbf{Z}_1^t\mathbf{Y}^{ZZ}\mathbf{Z}_1 + \mathbf{U}_1^t\mathbf{Y}^{UU}\mathbf{U}_1 + \mathbf{Z}_1^t\mathbf{Y}^{ZU}\mathbf{U}_1 \tag{S50}$$

with,

$$\mathbf{Y}^0 = \mu_\rho^{gk,0}\mu_\sigma^{kg,0} + (\mu_\rho^{gk,0}(\boldsymbol{\mu}_\sigma^{(1)})^t + \mu_\sigma^{kg,0}(\boldsymbol{\mu}_\rho^{(1)})^t)\mathbf{D}_{HT} + \mathrm{Tr}[\mathbf{M}_{\rho\sigma}\mathbf{D}_{HT}\mathbf{D}_{HT}^t] \tag{S51a}$$

$$\mathbf{Y}^Z = \frac{\sqrt{2}}{2}\left[\mu_\rho^{gk,0}(\boldsymbol{\mu}_\sigma^{(1)})^t + \mu_\sigma^{kg,0}(\boldsymbol{\mu}_\rho^{(1)})^t + \mathbf{D}_{HT}(\mathbf{M}_{\sigma\rho} + \mathbf{M}_{\rho\sigma})\right]\mathbf{D}^{-1/2} \tag{S51b}$$

$$\mathbf{Y}^U = \frac{\sqrt{2}}{2}\left[\mu_\rho^{gk,0}(\boldsymbol{\mu}_\sigma^{(1)})^t + \mu_\sigma^{kg,0}(\boldsymbol{\mu}_\rho^{(1)})^t - \mathbf{D}_{HT}(\mathbf{M}_{\sigma\rho} - \mathbf{M}_{\rho\sigma})\right]\mathbf{C}^{-1/2} \tag{S51c}$$

$$\mathbf{Y}^{ZZ} = \frac{1}{2}\mathbf{D}^{-1/2}\mathbf{M}_{\rho\sigma}\mathbf{D}^{-1/2} \tag{S51d}$$

$$\mathbf{Y}^{UU} = -\frac{1}{2}\mathbf{C}^{-1/2}\mathbf{M}_{\rho\sigma}\mathbf{C}^{-1/2} \tag{S51e}$$

$$\mathbf{Y}^{ZU} = \frac{1}{2}\mathbf{D}^{-1/2}(\mathbf{M}_{\sigma\rho} - \mathbf{M}_{\rho\sigma})\mathbf{C}^{-1/2} \tag{S51f}$$

where we have introduced the matrix $\mathbf{M}_{\sigma\rho}$ with elements $(M_{\sigma\rho})_{ij} = (\mu_\sigma^{(1)})_i(\mu_\rho^{(1)})_j$.

Before integrating, we also need to specify the type of bands (Rayleigh or fundamentals). Each type is revised in the following subsections.

S3.2.1 Rayleigh band

For the Rayleigh band, $F_{v_f^g} = 1$, and the correlation functions reads,

$$\begin{aligned}\chi_{\rho\sigma}^{|\mathbf{0}^g\rangle} &= \chi_0\frac{1}{(2\pi)^N}\int_{-\infty}^{\infty}\int_{-\infty}^{\infty} d\mathbf{Z}_1 d\mathbf{U}_1 \exp\left[-\frac{1}{2}\mathbf{Z}_1^t\mathbf{Z}_1\right]\exp\left[-\frac{1}{2}\mathbf{U}_1^t\mathbf{U}_1\right] \\ &\quad \left(\mathbf{Y}^0 + \mathbf{Y}^Z\mathbf{Z}_1 + \mathbf{Y}^U\mathbf{U}_1 + \mathbf{Z}_1^t\mathbf{Y}^{ZZ}\mathbf{Z}_1 + \mathbf{U}_1^t\mathbf{Y}^{UU}\mathbf{U}_1 + \mathbf{Z}_1^t\mathbf{Y}^{ZU}\mathbf{U}_1\right) \\ &= \chi_0\frac{1}{(2\pi)^N}\int_{-\infty}^{\infty}\int_{-\infty}^{\infty} d\mathbf{Z}_1 d\mathbf{U}_1 \exp\left[-\frac{1}{2}\mathbf{Z}_1^t\mathbf{Z}_1\right]\exp\left[-\frac{1}{2}\mathbf{U}_1^t\mathbf{U}_1\right] \\ &\quad \left(\mathbf{Y}^0 + \mathbf{Z}_1^t\mathbf{Y}^{ZZ}\mathbf{Z}_1 + \mathbf{U}_1^t\mathbf{Y}^{UU}\mathbf{U}_1\right)\end{aligned} \tag{S52}$$

where we take into account that only integrals with even exponents on $Z_{1,i}$ and $U_{1,i}$ are non-vanishing. Further using the expression for Gaussian integrals,

$$\int_{-\infty}^{\infty} dx\ e^{x^2/2} = \sqrt{2\pi} \tag{S53a}$$

$$\int_{-\infty}^{\infty} dx\ x^n e^{x^2/2} = (1 \cdot 3 \cdots (n-1))\sqrt{2\pi} \quad (n > 0 \text{ and even}) \tag{S53b}$$

the above integral can be fully evaluated leading to,

$$\begin{aligned}\chi_{\rho\sigma}^{|\mathbf{0}^g\rangle}(t,T) = \quad & \chi_0 \left[\mu_\rho^{gm,(0)} \mu_\sigma^{gm,(0)} + \mu_\rho^{gm,(0)} \sum_{i=1}^{N_{vib}} \mu_{\sigma,i}^{gm,(1)} (D_{HT})_i + \right. \\ & \left. \mu_\sigma^{gm,(0)} \sum_i \mu_{\rho,i}^{gm,(1)} (D_{HT})_i + \sum_{i,j} \mu_{\rho,i}^{gm,(1)} \mu_{\sigma,j}^{gm,(1)} (A_{HT})_{ji} \right]\end{aligned} \tag{S54}$$

where the sums run over the N_{vib} normal mode coordinates and we introduce the $\mathbf{A}_{HT}$ matrix as,

$$\mathbf{A}_{HT} = \mathbf{D}_{HT}\mathbf{D}_{HT}^t + \frac{1}{2}(\mathbf{D}^{-1} - \mathbf{C}^{-1}) \tag{S55}$$

S3.2.2 Fundamental bands

In the case of the k-th fundamental,

$$F_{v_f^g}(\mathbf{Q}_f) = \sqrt{2\Gamma_{ik}}(\boldsymbol{\alpha}\mathbf{Q}_f + K_k) = \sqrt{2\Gamma_{ik}} \left(\frac{\sqrt{2}}{2}\boldsymbol{\alpha}^t\mathbf{D}^{-1/2}\mathbf{Z}_1 + \frac{\sqrt{2}}{2}\boldsymbol{\alpha}^t\mathbf{C}^{-1/2}\mathbf{U}_1 + \boldsymbol{\alpha}^t\mathbf{D}_{HT} + K_k \right) \tag{S56}$$

and the correlation function reads,

$$\begin{aligned}\chi_{\rho\sigma}^{|\mathbf{0}^g+1_k\rangle} = \sqrt{2\Gamma_{ik}}\chi_0 \frac{1}{(2\pi)^N} \int_{-\infty}^{\infty}\int_{-\infty}^{\infty} & d\mathbf{Z}_1 d\mathbf{U}_1 \exp\left[-\frac{1}{2}\mathbf{Z}_1^t\mathbf{Z}_1\right] \exp\left[-\frac{1}{2}\mathbf{U}_1^t\mathbf{U}_1\right] \\ & \left(\frac{\sqrt{2}}{2}\boldsymbol{\alpha}^t\mathbf{D}^{-1/2}\mathbf{Z}_1 + \frac{\sqrt{2}}{2}\boldsymbol{\alpha}^t\mathbf{C}^{-1/2}\mathbf{U}_1 + \boldsymbol{\alpha}^t\mathbf{D}_{HT} + K_k \right) \\ & \left(\mathbf{Y}^0 + \mathbf{Y}^Z\mathbf{Z}_1 + \mathbf{Y}^U\mathbf{U}_1 + \mathbf{Z}_1^t\mathbf{Y}^{ZZ}\mathbf{Z}_1 + \mathbf{U}_1^t\mathbf{Y}^{UU}\mathbf{U}_1 + \mathbf{Z}_1^t\mathbf{Y}^{ZU}\mathbf{U}_1 \right)\end{aligned} \tag{S57}$$

again, we take into account that only integrals with even exponents on $Z_{1,i}$ and $U_{1,i}$ are non-vanishing. Further operating all the resulting Gaussian, we arrive to,

$$\chi_{\rho\sigma}^{|\mathbf{0}^g+1_k\rangle}(t,T) = \chi_0\sqrt{2\Gamma_{gk}}\Bigg[\mu_\rho^{gm,(0)}\mu_\sigma^{gm,(0)}\Bigg(K_k - \sum_i J_{ki}(D_{HT})_i\Bigg) +$$
$$\mu_\rho^{gm,(0)}\Bigg(K_k\sum_i \mu_{\sigma,i}^{gm,(1)}(D_{HT})_i - \sum_{i,j} J_{ki}\mu_{\sigma,j}^{gm,(1)}\Big[(D_{HT})_i(D_{HT})_j - \frac{1}{2}((D^{-1})_{ji} - (C^{-1})_{ji})\Big]\Bigg) +$$
$$\mu_\sigma^{gm,(0)}\Bigg(K_k\sum_i \mu_{\rho,i}^{gm,(1)}(D_{HT})_i - \sum_{i,j} J_{ki}\mu_{\rho,j}^{gm,(1)}\Big[(D_{HT})_i(D_{HT})_j - \frac{1}{2}((D^{-1})_{ji} + (C^{-1})_{ji})\Big]\Bigg) +$$
$$\sum_{ij}\mu_{\rho,i}^{gm,(1)}\mu_{\sigma,j}^{gm,(1)}\Bigg(K_k(A_{HT})_{ji} - (A_{HT})_{ji}\sum_l J_{kl}(D_{HT})_l +$$
$$\frac{1}{2}(D_{HT})_j\sum_l J_{kl}((D^{-1})_{il} + (C^{-1})_{il}) + \frac{1}{2}(D_{HT})_i\sum_l J_{kl}((D^{-1})_{jl} - (C^{-1})_{jl})\Bigg)\Bigg] \quad \text{(S58)}$$

S3.2.3 Overtones

We follow the same strategy to derive an analytical expression for overtones including HT terms. In this case, the final vibrational state has two quanta over a given normal mode, so that,

$$\langle \mathbf{0}^g + 2_k^g|\mathbf{Q}_i\rangle = \sqrt{2}\Gamma_{ik}(Q_{ik}^2 - 1/2\Gamma_{ik})\frac{\det[\mathbf{\Gamma}_i]}{\pi^{N/4}}e^{-\frac{1}{2}\mathbf{Q}_i^t\mathbf{\Gamma}_i\mathbf{Q}_i} \quad \text{(S59)}$$

Therefore, the corresponding $F_{v_f^g}$ factor is given by,

$$F_{v_f^g}(\mathbf{Q}_f) = \sqrt{2}\Gamma_{ik}\left[(\boldsymbol{\alpha}\mathbf{Q}_f + K_k)^2 - 1/2\Gamma_{ik}\right] = \sqrt{2}\Gamma_{ik}\left[\mathbf{X}^0 + \mathbf{X}^Z\mathbf{Z}_1 + \mathbf{X}^U\mathbf{U}_1 + \mathbf{Z}_1^t\mathbf{X}^{ZZ}\mathbf{Z}_1 + \mathbf{U}_1^t\mathbf{X}^{UU}\mathbf{U}_1 + \mathbf{Z}_1^t\mathbf{X}^{ZU}\mathbf{U}_1\right] \quad \text{(S60)}$$

were we introduce the $\mathbf{X}$ matrices to compactly write the expression, which are given by,

$$\mathbf{X}^0 = K_k^2 - 1/2\Gamma_{ik} + 2K_k\boldsymbol{\alpha}\mathbf{D}_{HT} + \mathrm{Tr}[\boldsymbol{\beta}\mathbf{D}_{HT}\mathbf{D}_{HT}^t] \quad \text{(S61a)}$$

$$\mathbf{X}^Z = \sqrt{2}(K_k\boldsymbol{\alpha}^t + \mathbf{D}_{HT}^t\boldsymbol{\beta})\mathbf{D}^{-1/2} \quad \text{(S61b)}$$

$$\mathbf{X}^{U} = \sqrt{2}(K_k\boldsymbol{\alpha}^t + \mathbf{D}_{HT}^t\boldsymbol{\beta})\mathbf{C}^{-1/2} \tag{S61c}$$

$$\mathbf{X}^{ZZ} = \frac{1}{2}\mathbf{D}^{-1/2}\boldsymbol{\beta}\mathbf{D}^{-1/2} \tag{S61d}$$

$$\mathbf{X}^{UU} = \frac{1}{2}\mathbf{C}^{-1/2}\boldsymbol{\beta}\mathbf{C}^{-1/2} \tag{S61e}$$

$$\mathbf{X}^{ZU} = \mathbf{D}^{-1/2}\boldsymbol{\beta}\mathbf{C}^{-1/2} \tag{S61f}$$

Using both the auxiliary $\mathbf{X}$ and $\mathbf{Y}$ matrices (see Eqs. S51), the correlation function can be compactly written as,

$$\begin{aligned}\chi = \sqrt{2}\Gamma_{ik}\chi_0\frac{1}{(2\pi)^N}\int_{-\infty}^{\infty}\int_{-\infty}^{\infty} d\mathbf{Z}_1 d\mathbf{U}_1 \exp\left[-\frac{1}{2}\mathbf{Z}_1^t\mathbf{Z}_1\right]\exp\left[-\frac{1}{2}\mathbf{U}_1^t\mathbf{U}_1\right]\{\mathbf{X}^0\mathbf{Y}^0 + \\ \mathbf{X}^0\mathbf{Z}_1^t\mathbf{Y}^{ZZ}\mathbf{Z}_1 + \mathbf{X}^0\mathbf{U}_1^t\mathbf{Y}^{UU}\mathbf{U}_1 + \mathbf{Y}^0\mathbf{Z}_1^t\mathbf{X}^{ZZ}\mathbf{Z}_1 + \mathbf{Y}^0\mathbf{U}_1^t\mathbf{X}^{UU}\mathbf{U}_1 + \\ \mathbf{Z}_1^t(\mathbf{X}^Z)^t\mathbf{Y}^Z\mathbf{Z}_1 + \mathbf{U}_1^t(\mathbf{X}^U)^t\mathbf{Y}^U\mathbf{U}_1 + \\ \mathbf{Z}_1^t\mathbf{X}^{ZZ}\mathbf{Z}_1\mathbf{Z}_1^t\mathbf{Y}^{ZZ}\mathbf{Z}_1 + \mathbf{U}_1^t\mathbf{X}^{UU}\mathbf{U}_1\mathbf{U}_1^t\mathbf{Y}^{UU}\mathbf{U}_1 + \\ \mathbf{Z}_1^t\mathbf{X}^{ZZ}\mathbf{Z}_1\mathbf{U}_1^t\mathbf{Y}^{UU}\mathbf{U}_1 + \mathbf{U}_1^t\mathbf{X}^{UU}\mathbf{U}_1\mathbf{Z}_1^t\mathbf{Y}^{ZZ}\mathbf{Z}_1 + \mathbf{Z}_1^t\mathbf{X}^{ZU}\mathbf{U}_1\mathbf{Z}_1^t\mathbf{Y}^{ZU}\mathbf{U}_1\}\end{aligned} \tag{S62}$$

The above integral can be solved taking into account the basic rules of Gaussian integration (Eqs. S53), leading to,

$$\begin{aligned}\chi = \sqrt{2}\Gamma_{ik}\chi_0\{\mathbf{X}^0\mathbf{Y}^0 + \mathrm{Tr}\left[(\mathbf{X}^Z)^t\mathbf{Y}^Z\right] + \mathrm{Tr}\left[(\mathbf{X}^U)^t\mathbf{Y}^U\right] + \mathbf{X}^0\mathrm{Tr}\left[\mathbf{Y}^{ZZ}\right] + \\ \mathbf{X}^0\mathrm{Tr}\left[\mathbf{Y}^{UU}\right] + \mathbf{Y}^0\mathrm{Tr}\left[\mathbf{X}^{ZZ}\right] + \mathbf{Y}^0\mathrm{Tr}\left[\mathbf{X}^{UU}\right] + \mathrm{Tr}\left[\mathbf{X}^{ZZ}\right]\mathrm{Tr}\left[\mathbf{Y}^{ZZ}\right] + \mathrm{Tr}\left[\mathbf{X}^{ZZ}(\mathbf{Y}^{ZZ})^t\right] + \\ \mathrm{Tr}\left[\mathbf{X}^{ZZ}\mathbf{Y}^{ZZ}\right] + \mathrm{Tr}\left[\mathbf{X}^{UU}\right]\mathrm{Tr}\left[\mathbf{Y}^{UU}\right] + \mathrm{Tr}\left[\mathbf{X}^{UU}(\mathbf{Y}^{UU})^t\right] + \mathrm{Tr}\left[\mathbf{X}^{UU}\mathbf{Y}^{UU}\right] + \\ \mathrm{Tr}\left[\mathbf{X}^{ZZ}\right]\mathrm{Tr}\left[\mathbf{Y}^{UU}\right] + \mathrm{Tr}\left[\mathbf{X}^{UU}\right]\mathrm{Tr}\left[\mathbf{Y}^{ZZ}\right] + \mathrm{Tr}\left[\mathbf{X}^{ZU}(\mathbf{Y}^{ZU})^t\right]\}\end{aligned} \tag{S63}$$

Substituting the actual expressions for $\mathbf{X}$ and $\mathbf{Y}$ matrices, we arrive to the final expression corresponding to the correlation function for RR overtones (in matrix notation),

$$
\begin{aligned}
\chi_{\rho\sigma}^{|\mathbf{0}^g+2_k\rangle} = \sqrt{2}\Gamma_{ik}\chi_0 \{ \\
\left(\boldsymbol{\mu}_\rho^0\boldsymbol{\mu}_\sigma^0 + (\boldsymbol{\mu}_\rho^0\boldsymbol{\mu}'_\sigma + \boldsymbol{\mu}'_\rho\boldsymbol{\mu}_\sigma^0)\mathbf{D}_{HT} + \mathrm{Tr}[\mathbf{M}_{\rho\sigma}\mathbf{A}_{HT}]\right)\left(K_k^2 - 1/2\Gamma_{ik} + 2K_k\boldsymbol{\alpha}^t\mathbf{D}_{HT} + \mathrm{Tr}[\boldsymbol{\beta}\mathbf{B}_{HT}]\right) + \\
(K_k + \boldsymbol{\alpha}^t\mathbf{D_{HT}})\left(\boldsymbol{\mu}_\sigma^0\mathrm{Tr}[(\mathbf{D}^{-1}+\mathbf{C}^{-1})\boldsymbol{\alpha}\boldsymbol{\mu}'_\rho] + \boldsymbol{\mu}_\rho^0\mathrm{Tr}[(\mathbf{D}^{-1}-\mathbf{C}^{-1})\boldsymbol{\alpha}\boldsymbol{\mu}'_\sigma] + \right. \\
\left. \mathrm{Tr}[(\mathbf{D}^{-1}+\mathbf{C}^{-1})\boldsymbol{\alpha}\mathbf{D}_{HT}\mathbf{M}_{\sigma\rho}] + \mathrm{Tr}[(\mathbf{D}^{-1}-\mathbf{C}^{-1})\boldsymbol{\alpha}\mathbf{D}_{HT}\mathbf{M}_{\rho\sigma}]\right) + \\
\frac{1}{2}\mathrm{Tr}[(\mathbf{D}^{-1}-\mathbf{C}^{-1})\boldsymbol{\beta}(\mathbf{D}^{-1}+\mathbf{C}^{-1})\mathbf{M}_{\sigma\rho}]\}
\end{aligned}
\tag{S64}
$$

where we introduced the matrix $\mathbf{B}_{HT} = \mathbf{D}_{HT}\mathbf{D}_{HT}^t + \frac{1}{2}(\mathbf{D}^{-1} + \mathbf{C}^{-1})$.

S3.2.4 Combination bands

In combination bands, the final vibrational state has one quanta over tow different normal modes, so that,

$$
\langle\mathbf{0}^g + 1_k^g + 1_l^g|\mathbf{Q}_i\rangle = 2\sqrt{\Gamma_{ik}\Gamma_{il}}Q_{ik}Q_{il}\frac{\det[\boldsymbol{\Gamma}_i]}{\pi^{N/4}}e^{-\frac{1}{2}\mathbf{Q}_i^t\boldsymbol{\Gamma}_i\mathbf{Q}_i} \tag{S65}
$$

Therefore, the corresponding $F_{v_f^g}$ factor is given by,

$$
\begin{aligned}
F_{v_f^g}(\mathbf{Q}_f) = 2\sqrt{\Gamma_{ik}\Gamma_{il}}(\boldsymbol{\alpha}_k\mathbf{Q}_f + K_k)(\boldsymbol{\alpha}_l\mathbf{Q}_f + K_l) = \\
2\sqrt{\Gamma_{ik}\Gamma_{il}}\left[\mathbf{X}^0 + \mathbf{X}^Z\mathbf{Z}_1 + \mathbf{X}^U\mathbf{U}_1 + \right. \\
\left. \mathbf{Z}_1^t\mathbf{X}^{ZZ}\mathbf{Z}_1 + \mathbf{U}_1^t\mathbf{X}^{UU}\mathbf{U}_1 + \mathbf{Z}_1^t\mathbf{X}^{ZU}\mathbf{U}_1\right]
\end{aligned}
\tag{S66}
$$

were we differenciate the $\boldsymbol{\alpha}$ vectors as the corresponding (k and l) rows of the Duschinsky matrix. Similarly, we build different $\boldsymbol{\beta}$ matrices as $\boldsymbol{\beta}_{kl} = \boldsymbol{\alpha}_k\boldsymbol{\alpha}_l^t$. The expressions for the auxiliar $\mathbf{X}$ matrices now read,

$$
\mathbf{X}^0 = K_kK_l + (K_k\boldsymbol{\alpha}_l + K_l\boldsymbol{\alpha}_k)\mathbf{D}_{HT} + \mathrm{Tr}[\boldsymbol{\beta}_{kl}\mathbf{D}_{HT}\mathbf{D}_{HT}^t] \tag{S67a}
$$

$$
\mathbf{X}^Z = \frac{\sqrt{2}}{2}\left[(K_k\boldsymbol{\alpha}_l^t + K_l\boldsymbol{\alpha}_k^t) + \mathbf{D}_{HT}^t(\boldsymbol{\beta}_{kl} + \boldsymbol{\beta}_{lk})\right]\mathbf{D}^{-1/2} \tag{S67b}
$$

$$
\mathbf{X}^U = \frac{\sqrt{2}}{2}\left[(K_k\boldsymbol{\alpha}_l^t + K_l\boldsymbol{\alpha}_k^t) + \mathbf{D}_{HT}^t(\boldsymbol{\beta}_{kl} + \boldsymbol{\beta}_{lk})\right]\mathbf{C}^{-1/2} \tag{S67c}
$$

$$
\mathbf{X}^{ZZ} = \frac{1}{2}\mathbf{D}^{-1/2}\boldsymbol{\beta}_{kl}\mathbf{D}^{-1/2} \tag{S67d}
$$

$$\mathbf{X}^{UU} = \frac{1}{2}\mathbf{C}^{-1/2}\boldsymbol{\beta}_{kl}\mathbf{C}^{-1/2} \tag{S67e}$$

$$\mathbf{X}^{ZU} = \mathbf{D}^{-1/2}(\boldsymbol{\beta}_{kl} + \boldsymbol{\beta}_{lk})\mathbf{C}^{-1/2} \tag{S67f}$$

Again, we get to a similar general solution in terms of $\mathbf{X}$ and $\mathbf{Y}$ matrices, which finally leads to the following expression (in matrix notation),

$$\begin{aligned}
\chi_{\rho\sigma}^{|\mathbf{0}^g+1_k+1_l\rangle} = 2\sqrt{\Gamma_{ik}\Gamma_{il}}\chi_0 \{ \\
\left(\boldsymbol{\mu}^0_\rho\boldsymbol{\mu}^0_\sigma + (\boldsymbol{\mu}^0_\rho\boldsymbol{\mu}'_\sigma + \boldsymbol{\mu}'_\rho\boldsymbol{\mu}^0_\sigma)\mathbf{D}_{HT} + \mathrm{Tr}[\mathbf{M}_{\rho\sigma}\mathbf{A}_{HT}]\right)\left(K_kK_l + (K_k\boldsymbol{\alpha}_l + K_l\boldsymbol{\alpha}_k)\mathbf{D}_{HT} + \mathrm{Tr}[\boldsymbol{\beta}_{kl}\mathbf{B}_{HT}]\right) + \\
\frac{1}{2}(K_k + \boldsymbol{\alpha}_k^t\mathbf{D_{HT}})\left(\boldsymbol{\mu}^0_\sigma\mathrm{Tr}[(\mathbf{D}^{-1} + \mathbf{C}^{-1})\boldsymbol{\alpha}_l\boldsymbol{\mu}'_\rho] + \boldsymbol{\mu}^0_\rho\mathrm{Tr}[(\mathbf{D}^{-1} - \mathbf{C}^{-1})\boldsymbol{\alpha}_l\boldsymbol{\mu}'_\sigma] + \right. \\
\left.\mathrm{Tr}[(\mathbf{D}^{-1} + \mathbf{C}^{-1})\boldsymbol{\alpha}_l\mathbf{D}_{HT}\mathbf{M}_{\sigma\rho}] + \mathrm{Tr}[(\mathbf{D}^{-1} - \mathbf{C}^{-1})\boldsymbol{\alpha}_l\mathbf{D}_{HT}\mathbf{M}_{\rho\sigma}]\right) + \\
\frac{1}{2}(K_l + \boldsymbol{\alpha}_l^t\mathbf{D_{HT}})\left(\boldsymbol{\mu}^0_\sigma\mathrm{Tr}[(\mathbf{D}^{-1} + \mathbf{C}^{-1})\boldsymbol{\alpha}_k\boldsymbol{\mu}'_\rho] + \boldsymbol{\mu}^0_\rho\mathrm{Tr}[(\mathbf{D}^{-1} - \mathbf{C}^{-1})\boldsymbol{\alpha}_k\boldsymbol{\mu}'_\sigma] + \right. \\
\left.\mathrm{Tr}[(\mathbf{D}^{-1} + \mathbf{C}^{-1})\boldsymbol{\alpha}_k\mathbf{D}_{HT}\mathbf{M}_{\sigma\rho}] + \mathrm{Tr}[(\mathbf{D}^{-1} - \mathbf{C}^{-1})\boldsymbol{\alpha}_k\mathbf{D}_{HT}\mathbf{M}_{\rho\sigma}]\right) + \\
\frac{1}{4}\mathrm{Tr}[(\mathbf{D}^{-1} - \mathbf{C}^{-1})\boldsymbol{\beta}_{kl}(\mathbf{D}^{-1} + \mathbf{C}^{-1})\mathbf{M}_{\sigma\rho}] + \frac{1}{4}\mathrm{Tr}[(\mathbf{D}^{-1} + \mathbf{C}^{-1})\boldsymbol{\beta}_{kl}(\mathbf{D}^{-1} - \mathbf{C}^{-1})\mathbf{M}_{\rho\sigma}]\}
\end{aligned} \tag{S68}$$

The resulting expression for overtones and combination bands involve a considerable increased computational cost, mainly at HT level. The modes involved can be selected from input to limit the computational burden. By default, all overtones are computed, while for combinations one could fist run a calculation with all fundamentals and account for the combinations of only those modes with the more intense fundamentals. Other approximative strategies to reduce the computational based on generalized thresholds to identify zero elements in the Duschinsky and dipole derivatives vectors have also been proposed[10]. In our implementaton, we compute all tensor elements simultaneously, and avoid recomputing some arrays that have the same value among iterations. This requires some extra memory, but significantly accelerates the calculations. This strategy can be generalized to group different modes that are computed simultaneously.

S3.3 Broadening functions

In order to account for additional broadening effects, such as solvent inhomogeneous broadening or natural broadening, the spectra can be convoluted with a convenient broadening function (Gaussian, Lorentzian or a combination of them) that account for such

effects. TD spectra achieve this by multiplying the correlation function by the corresponding decay function, corresponding to the Fourier-transform of the related broadening function in the frequency domain. Other types of broadening, namely exponential decays (right, R, or left, L) are also implemented within the `reconvolute_TD` tool. The corresponding expressions in frequency and time domain for each type of broadening are given below,

Broad.	Freq. domain	Time domain	Rel. with HWHM
Gaussian	$\frac{1}{\sigma\sqrt{2\pi}}e^{\omega^2/2\sigma^2}$	$\frac{1}{\sqrt{2\pi}}e^{\sigma^2 t^2/2}$	$\sigma = \frac{\text{HWHM}}{\sqrt{2\ln 2}}$
Lorentzian	$\frac{1}{\pi}\frac{\gamma}{\omega^2+\gamma^2}$	$\frac{1}{\sqrt{2\pi}}e^{-\gamma\lvert t\rvert}$	$\gamma = \text{HWHM}$
Exp. decay (R)	$ae^{-a\omega}$; $\omega \geq 0$	$\frac{1}{\sqrt{2\pi}}\frac{1}{a-it}$	$a = \frac{\ln 2}{2\text{HWHM}}$
Exp. decay (L)	$ae^{a\omega}$; $\omega \leq 0$	$\frac{1}{\sqrt{2\pi}}\frac{1}{a+it}$	$a = \frac{\ln 2}{2\text{HWHM}}$

As indicated above, both Gaussian and Lorentzian broadening are available in $\mathcal{FC}$*classes3*, which lead to Gaussian and exponential decays in the time domain. Additionally, both broadening can also be combined, which is sometimes referred to as a Voigt profile. In TI, this implies the successive applications of each broadening function, while, by virtue of the convolution theorem, it leads to simply multiplying the corresponding decays in the time domain. In all cases, the extension of the broadening is controlled by the width of the function, usually characterized by the Half Width at Half Maximum (HWHM).

S3.4 Discrete Fourier-transform of the correlation function

Although the analytical expression for the correlation function is known for different spectroscopies, its Fourier-transform (FT) must be evaluated numerically. This first requires substituting the infinite range for the integral by a finite range, $-\tau_0$ to τ_0. Since the correlation functions monotonically decay to zero for $t \to \pm\infty$, selecting a sufficiently sizable finite range would suffice to get reliable results. In any case, to reduce numerical

instabilities, we further applied a Hann window, which implies the multiplication of the correlation function by $\cos^2(\pi t/2\tau_0)$.

Taking the OPA/EMI lineshape as an example (other properties will follow equivalently), the lineshape, $L(\omega)$, is computed with the TD method according to Eq. (11) in the manuscript. The numerical evaluation of the integral requires the discretization of the time domain, i.e., $t_m = \tau_0 + m\Delta t$ $(m = 0, \ldots, N)$, where $\Delta t = 2\tau_0/N$. Moreover, the function in the frequency domain also needs to be discretized, with $\omega_k = k\Delta\omega$ $(k = 0, \ldots)$.

$$
\begin{aligned}
L(\omega_k) \quad &\approx \int_{-\tau_0}^{\tau_0} dt e^{\pm i\omega t} \underbrace{\left[e^{\mp it\Delta E/\hbar}\chi(t,T)g'(t)\right]}_{F(t)} = \Delta t \cdot e^{\mp i\omega_k\tau_0} \sum_{m=0}^{N-1} F(t_m) e^{\pm imk\Delta t\Delta\omega} \\
&= \Delta t \cdot e^{\mp i\omega_k\tau_0} \sum_{m=0}^{N-1} F(t_m) e^{\pm i2\pi mk\Delta t\Delta\nu} = \Delta t \cdot e^{\mp i\omega_k\tau_0} \sum_{m=0}^{N-1} F(t_m) e^{\pm i2\pi\frac{mk}{N}}
\end{aligned}
\tag{S69}
$$

where we have set $\Delta\nu$ so that $\Delta t\Delta\nu = 1/N$. For $\pm$ and $\mp$ symbols, upper one corresponds to absorption and lower one to emission.

The last sum can be very efficiently evaluated with Fast Fourier Transform techniques if the number of sampled points is a power of 2. The code can be compiled with the FFTW3 library, which provides efficient implementations of both FFT and general discrete FT. In any case, FT is not the bottleneck of the calculation, and it can also be evaluated with a less efficient routine included with the code.

S3.5 Spectral grid for TI and TD methods

In order to generate equivalent spectral grids for TI and TD calculations, the program always reads the limits of the output spectrum in the frequency domain, along with the desired resolution, $\Delta\omega$. In the case of TD spectra, the spectral range and resolution are controlled by Δt and N, which are computed to match the spectral limits given in the frequency domain. It should be noted that the frequency range resulting from the FT begins at $\omega = 0$. In order to obtain a spectrum starting from $\omega \neq 0$, the correlation function is multiplied by $e^{-i\Delta\epsilon t/\hbar}$ to achieve a shift of $\Delta\epsilon$ in the frequency range, which

is then restored on the final spectrum. The quality of the numerical integration can be inspected *a posteriori* by ensuring that the spectrum goes to zero at both the red a blue tails.

S4 Additional computations on the selected applications

S4.1 Systematic spectroscopic analysis of a rigid system

S4.1.1 OPA and EMI of anthracene

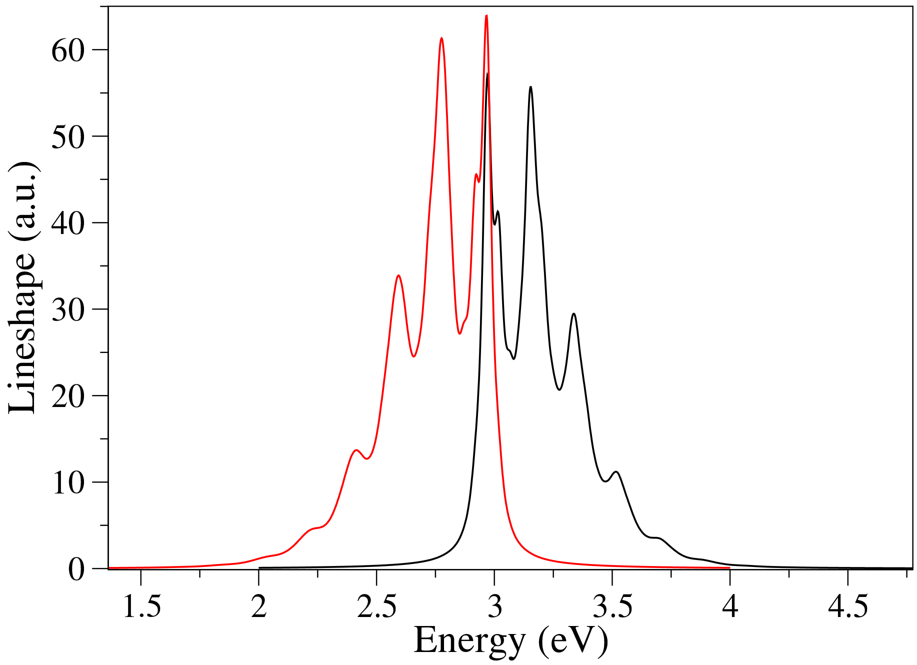

Figure S2: Absorption and emission lineshapes for S0→S1 transition at 300 K, simulated with FC|AH model (with Cartesian coordinates). Electronic data evaluated at B3LYP/6-31G(d) in the gas phase. A Lorentzian broadening with HWHM=0.02 eV was applied for all spectra.

S4.1.2 RR spectra of anthracene

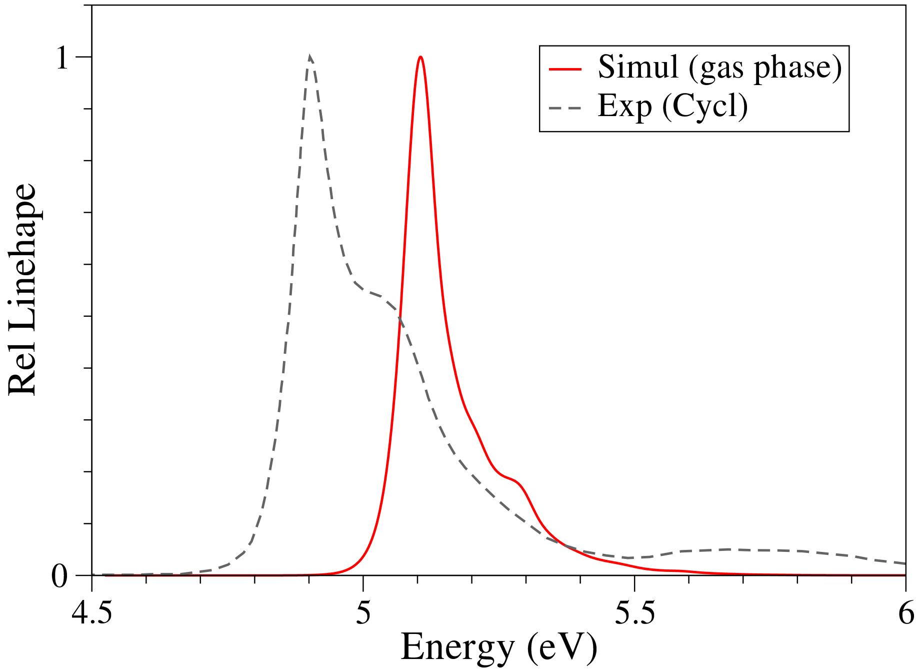

Figure S3: Absorption lineshape for S0→S5 transition simulated with HTi|VH model with electonic data at B3LYP/6-31G(d) in the gas phase. The experimental spectrum from ref. [20] is included for comparison.

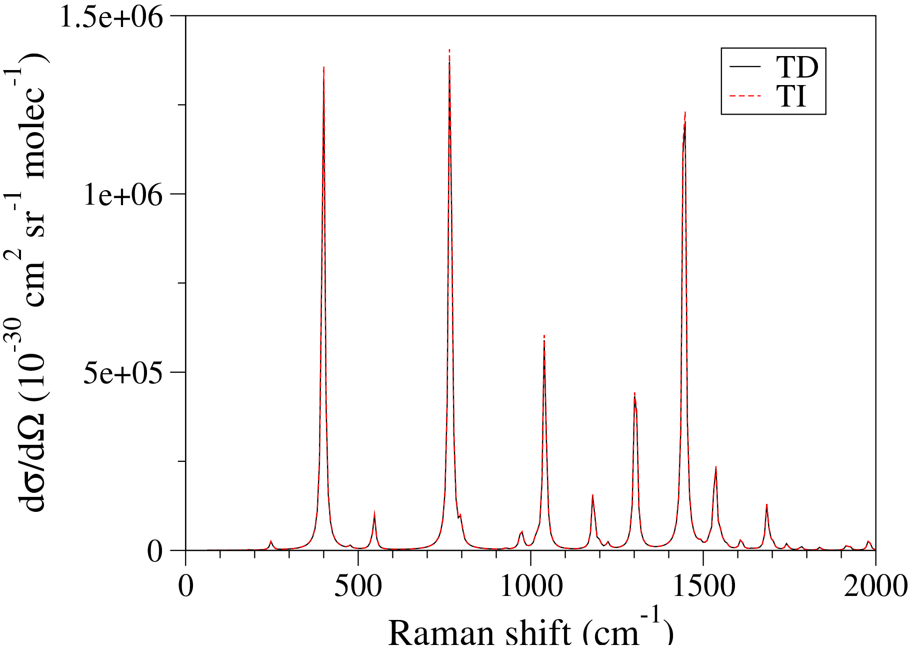

Figure S4: Resonce Raman spectra of anthracene with HTi|VH model including up to overtones for each normal mode. The calculation is carried out both with TI and TD methods with $\omega_I \approx 41130\,\mathrm{cm}^{-1}$.

Table S1: Description of the normal modes mainly involved in the RR spectrum of anthracene. Indices correspond to those adopted in the main text.

Index	Symm	Desciption
5	B_{2g}	Ring-place torsion
8	A_g	Accordion-like
10	B_{1g}	C-H out-of-plane bending
13	B_{2g}	C-H out-of-plane bending
42	A_g	CH in-plane rocking with CC stretching
48	A_g	CC stretching
51	A_g	CH in-plane rocking with CC stretching
55	B_{3g}	CC stretching

S4.2 OPA and EMI for flexible molecules

S4.2.1 Tuning imaginary frequency VH (EMI for p-Terphenyl)

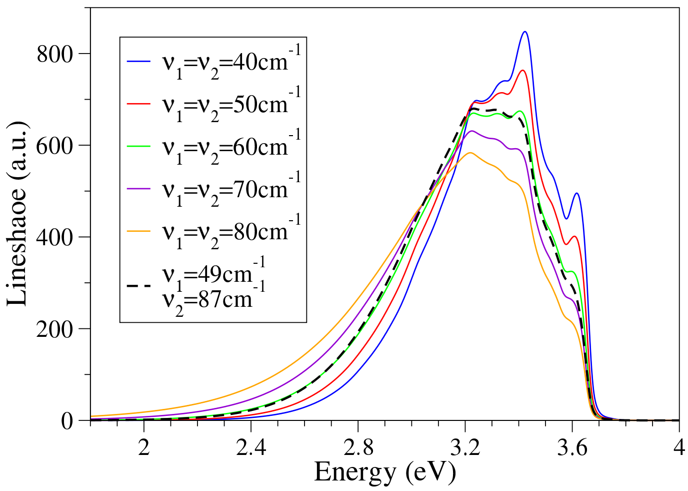

Figure S5: Emission spectra simulated at 300 K for p-terphenyl adopting VH. The two imaginary frequencies that arise (ν_1 and ν_2), related to the torsions, are set to different values, including those obtained turning the original imaginary values to real (dashed line).

S4.2.2 Energy scan along flexible torsions in polyphenyl molecules

In order to evaluate the coupling between the torsional profiles for p-terphenyl, the relaxed scan is computed for one torsion, either relaxing the other one or keeping it fixed at an intermediate value (60 degrees). The results in the table below indicate that while S0 profiles are not very sensitive to the position of the other dihedral (i.e. they are mostly uncoupled), some differences are observed at S1, which indicates that there is a non-negligible but still moderate coupling.

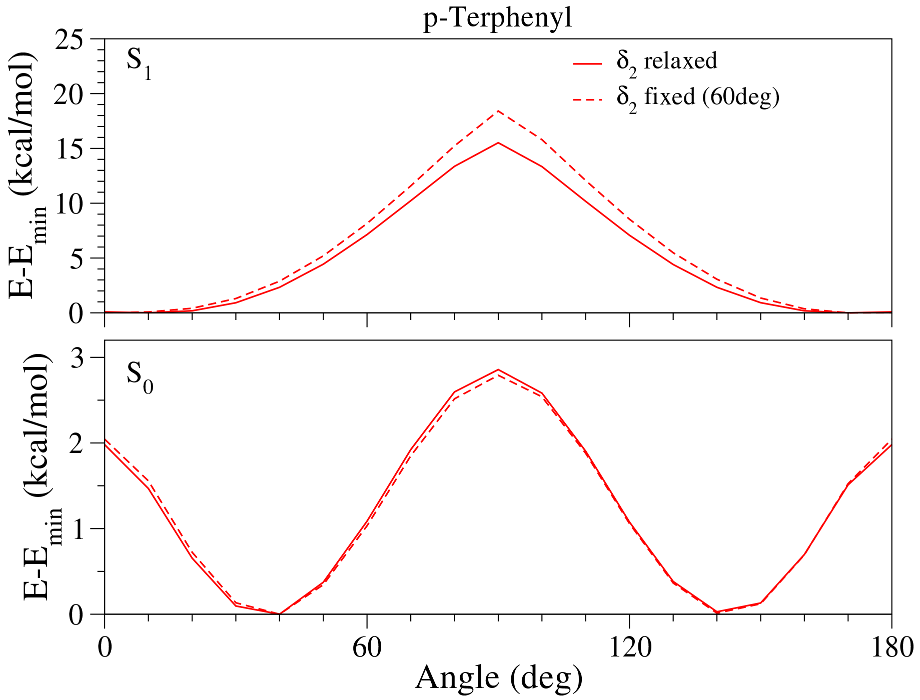

Figure S6: Relaxed potential energy curves corresponding to one flexible dihedral of p-terphenyl at PBE0/6-31G(d). S_1 corresponds to the bright H→L transition. The non-scanned dihedral is either relaxed at each iteration or keep fixed at 60 degrees in order to asses the coupling between both torsions.

S4.2.3 Effect of torsional distribution in the absorption spectral shape (polyphenyl)

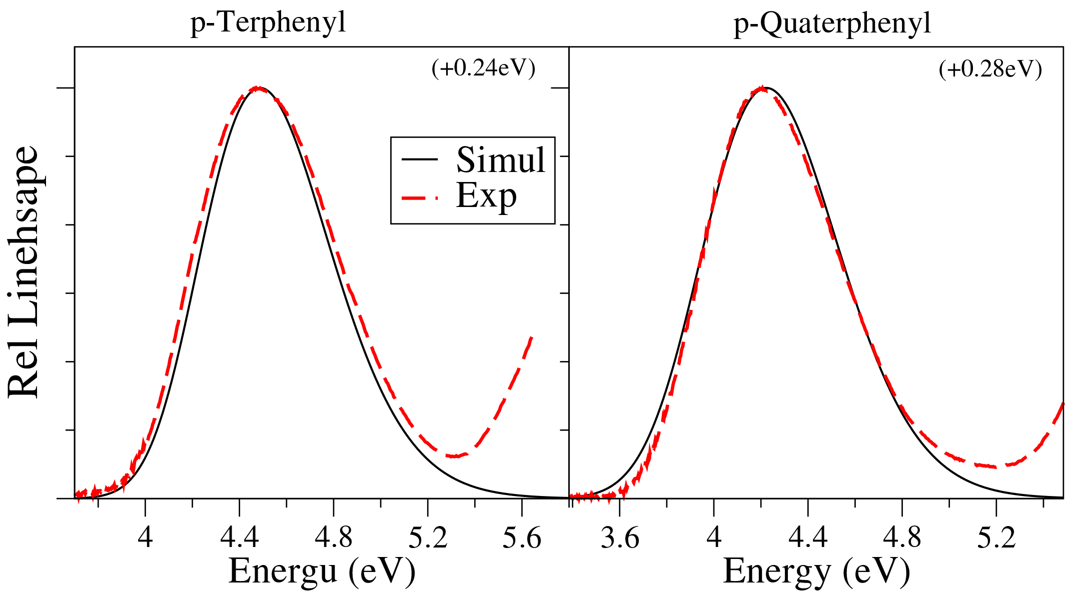

Figure S7: Absorption lineshape spectra simulated with the VH model using PBE0. The torsion is removed from the calculation and the effect is recovered by convolution with a Gaussian with the HWHM corresponding to the distribution of vertical energies over torsional space. All torsional profiles described with those of biphenyl.

References

[1] J. Cerezo, D. Aranda, F. J. Avila Ferrer, G. Prampolini, and F. Santoro, J. Chem. Theory Comput. **16**, 1215 (2020).

[2] N. Lin, H. Solheim, X. Zhao, F. Santoro, and K. Ruud, J. Chem. Theory Comput. **9**, 1557 (2013).

[3] N. Lin, F. Santoro, A. Rizzo, Y. Luo, X. Zhao, and V. Barone, J. Phys. Chem. A **113**, 4198 (2009).

[4] P. Macak, Y. Luo, and H. Ågren, Chem. Phys. Lett. **330**, 447 (2000).

[5] M.-L. Zhang and E. Pollak, J. Phys. Chem. A **109**, 122 (2005).

[6] H. Ma, Y. Zhao, and W. Liang, J. Chem. Phys. **140**, 094107 (2014).

[7] A. Baiardi, J. Bloino, and V. Barone, J. Chem. Phys. **141**, 114108 (2014).

[8] F. Santoro, C. Cappelli, and V. Barone, J. Chem. Theory Comput. **7**, 1824 (2011).

[9] H. Ma, J. Liu, and W. Liang, J. Chem. Theory Comput. **8**, 4474 (2012).

[10] B. de Souza, G. Farias, F. Neese, and R. Izsák, J. Chem. Phys. **150**, 214102 (2019).

[11] F. J. Avila Ferrer, V. Barone, C. Cappelli, and F. Santoro, J. Chem. Theory Comput. **9**, 3597 (2013).

[12] J. Cerezo, L. Martínez-Fernández, R. Improta, and F. Santoro, Theor. Chem. Acc. **135**, 221 (1 (2016).

[13] Y. Liu, D. Aranda, and F. Santoro, Phys. Chem. Chem. Phys. , (2021).

[14] Q. Peng, Y. Niu, C. Deng, and Z. Shuai, Chem. Phys. **370**, 215 (2010).

[15] R. Borrelli and A. Peluso, J. Chem. Theory Comput. **11**, 415 (2015).

[16] S. Banerjee, A. Baiardi, J. Bloino, and V. Barone, J. Chem. Theory Comput. **12**, 774 (2016).

[17] A. Humeniuk, M. Bužančić, J. Hoche, J. Cerezo, R. Mitrić, F. Santoro, and V. Bonačić-Koutecký, The Journal of Chemical Physics **152**, 054107 (2020).

[18] R. Borrelli, A. Capobianco, and A. Peluso, J. Phys. Chem. A **116**, 9934 (2012).

[19] J. von Cosel, J. Cerezo, D. Kern-Michler, C. Neumann, L. J. G. W. van Wilderen, J. Bredenbeck, F. Santoro, and I. Burghardt, J. Chem. Phys. **147**, 164116 (2017).

[20] T. Holtum, J. Bloino, C. Pappas, V. Kumar, V. Barone, and S. Schlücker, J. Raman Spectrosc. **52**, 2292 (2021).
